# Microbial Biogeography and Core Microbiota of the Rat Digestive Tract

**DOI:** 10.1038/srep45840

**Published:** 2017-04-04

**Authors:** Dongyao Li, Haiqin Chen, Bingyong Mao, Qin Yang, Jianxin Zhao, Zhennan Gu, Hao Zhang, Yong Q. Chen, Wei Chen

**Affiliations:** 1 State Key Laboratory of Food Science and Technology, School of Food Science and Technology, Jiangnan University, Wuxi 214122, P.R. China; 2 Departments of Cancer Biology and Biochemistry, Wake Forest School of Medicine, Winston-Salem, NC 27157, USA; 3 Beijing Innovation Centre of Food Nutrition and Human Health, Beijing Technology and Business University (BTBU), Beijing 100048, P.R. China

## Abstract

As a long-standing biomedical model, rats have been frequently used in studies exploring the correlations between gastrointestinal (GI) bacterial biota and diseases. In the present study, luminal and mucosal samples taken along the longitudinal axis of the rat digestive tract were subjected to 16S rRNA gene sequencing-based analysis to determine the baseline microbial composition. Results showed that the community diversity increased from the upper to lower GI segments and that the stratification of microbial communities as well as shift of microbial metabolites were driven by biogeographic location. A greater proportion of lactate-producing bacteria (such as Lactobacillus, Turicibacter and Streptococcus) were found in the stomach and small intestine, while anaerobic Lachnospiraceae and Ruminococcaceae, fermenting carbohydrates and plant aromatic compounds, constituted the bulk of the large-intestinal core microbiota where topologically distinct co-occurrence networks were constructed for the adjacent luminal and mucosal compartments. When comparing the GI microbiota from different hosts, we found that the rat microbial biogeography might represent a new reference, distinct from other murine animals. Our study provides the first comprehensive characterization of the rat GI microbiota landscape for the research community, laying the foundation for better understanding and predicting the disease-related alterations in microbial communities.

A large variety of symbiotic microorganisms coexist in the mammalian digestive tract, with their number around 10 times greater than the total number of mammalian somatic and germ cells^1^. They provide many functions that are not encoded in the host genome^2^. The microbiota inhabiting the digestive tract constitutes a complex ecosystem and plays a critical role in maintaining host physiological homeostasis^3^,^4^. In the last decade, with the rapid development of high-throughput sequencing technology, a large body of research has investigated the gut flora composition in humans^5^,^6^,^7^ and revealed its relationship to diseases^8^,^9^,^10^,^11^,^12^. However, most of the samples used in these studies were from feces^9^,^10^,^11^ and the appropriateness of using feces as a proxy for other GI segments remained questioned. On the other hand, because of ethical restrictions, microbiota profiling for given GI regions could only be found in a short list of studies, mainly through hospital-based endoscopic biopsies^7^,^13^,^14^.

Due to limitations in human research, murine models have become crucial in studies of the gut microbiota designed to obtain mechanism insights into different anatomical regions through radical and sometimes even destructive means. The rat is the first mammalian species to have been domesticated for scientific purposes. Initial foundation work began in the early 1900’s at the Wistar Institute of Philadelphia, where one of the first breeding experiments was recorded by Helen King^15^. As a long-standing model in biomedical research, rats have recently been used in numerous studies exploring the correlations between intestinal bacterial biota and various kinds of diseases, such as ulcerative colitis^16^, colon cancer^17^,^18^, mucositis^19^, gastric ulceration^20^, obesity^21^, diabetes^22^, hypertension^23^, anxiety^24^ and more. A comprehensive characterization of the normal rat microbiota landscape is a critical prerequisite to understanding and predicting disease-related alterations in microbial communities. However, our knowledge about the spatial structure of the microbial consortium in the rat is very limited.

The common intestinal symbiotic microorganisms usually act as key ecosystem service providers^25^ for mammals. Understanding their co-occurring interactions with each other and with their hosts is of great importance for microbiome research. However, the mammalian digestive system is composed of many different niches with distinct physicochemical conditions^26^,^27^,^28^,^29^. In the process of evolution, the commensal bacteria are selected by harsh intestinal environments and co-evolve with their hosts^30^. Facing the selection pressure and other challenges, microbes inhabiting different GI niches devoted extraordinary efforts to environmental adaption by employing distinct strategies, and in turn could provide indispensable ecosystem services at different anatomical sites. Amongst these, the mucus layer forms a distinct habitat with intimacy to host intestinal tissues, and the mucosal microbial metabolites are known to determine the differentiation and function of epithelial and immune cells^31^.

For these reasons, we have determined the baseline radial composition of taxa and microbial metabolites along the longitudinal axis of the rat digestive tract, analyzed the difference in community membership and structure and their relationship, unveiled the core microbiota and their co-occurrence relationships within different GI niches and compared the internal biogeographic maps of rats, other rodents as well as human beings. Our study aimed to answer the following questions: (i) How do microbial communities and metabolites change along the length of rat digestive tract? (ii) Which microbial assemblages inhabit each GI niche? Is there a core microbiota for a given GI region? (iii) Does the taxonomic profile in feces reflect that of other GI segments? (iv) Do similar physicochemical conditions in identical anatomical sites across host genotype backgrounds shape similar GI microbiota?

## Results

### Samples and Illumina sequencing

The average lengths of small intestines (including duodenum, jejunum and ileum) and colons from 6 seven-week-old rats were 67.83 ± 1.35 and 11.67 ± 0.33 cm, respectively. The relative mass of different gut segments varied, with the cecum being the largest digestive chamber (Supplemental Table S1).

Probably due to the low biomass of gastric and small-intestinal mucosally adherent microbiota, the extraction of microbial genomes in these mucus layers failed. Therefore, the sampling sites for each subject included gastric contents (Sto), duodenal contents (Duo), jejunal contents (Jej), ileal contents (Ile), cecal contents and mucus (Cec and Cem), proximal colonic contents and mucus (PCc and PCm), middle colonic contents and mucus (MCc and MCm), distal colonic contents and mucus (DCc and DCm) and feces (Fec). However, for subjects 1, 5 and 6, some colonic segments lacked luminal contents (Supplemental Table S2).

Illumina pair-end sequencing returned a total of 4,957,802 raw sequences across 75 samples from 6 rats. After assembly and quality filtering, a total of 4,009,159 sequences were used in downstream analyses. Each sample was covered by an average of 53,455 reads (ranging from 22,887 to 195,780). The Shannon diversity curves for all samples plateaued at current sequencing depth, while unfortunately the Chao1 curves failed to level off for most samples (Supplemental Fig. S1). This suggests that the current analysis has already captured most of the microbial diversity, although more phylotypes may be found by increasing the sequencing depth. Clustering at 97% identity produced 36,214 unique operational taxonomic units (OTUs) in total and 1,914 OTUs on average for each sample (ranging from 434 to 6,005). Good’s coverage of all samples averaged 98.1% (ranging from 96.1% to 99.3%) (Supplemental Table S2).

### Microbial community diversity estimates

**Figure 1:**
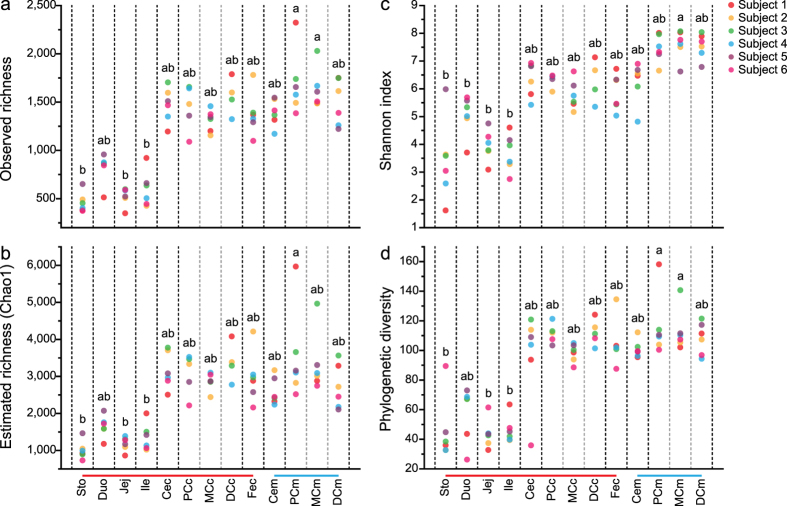
Diversity measurements for each sample. (**a**) Observed richness, (**b**) estimated richness, (**c**) Shannon index and (**d**) phylogenetic diversity measurements at each anatomic site from the 6 rats, where each point represents a sample. Ten iterations of rarefied subsets of 22,880 sequences from each sample were used to calculate the average values for these metrics. Different letters (**a**,**b**) above the points mean significant differences between sampling sites (Nemenyi’s *post hoc* multiple comparison test, FDR < 0.05). Sto: gastric contents; Duo: duodenal contents; Jej: jejunal contents; Ile: ileal contents; Cec: cecal contents; PCc: proximal colonic contents; MCc: middle colonic contents DCc: distal colonic contents; Fec: feces; Cem: cecal mucus; PCm: proximal colonic mucus; MCm: middle colonic mucus; DCm: distal colonic mucus. The red bar at the bottom of the graphs is used to highlight luminal samples, and the blue line highlights mucosal samples.

The observed and estimated species richness, Shannon index (SI) and phylogenetic diversity (PD) were used to evaluate the community diversity for each sample (Fig. 1). Generally speaking, richness and diversity was lower in gastric and small-intestinal samples than in large-intestinal samples. Unexpectedly, given the relatively low biomass in this region, samples from the colonic mucus layer had the highest richness and diversity. In addition, the SI values of gastric samples showed much higher inter-subject variation. On the opposite side of the spectrum, Shannon diversity in colonic mucus layers was much more consistent across subjects than in any of the other sites tested.

Manichanh *et al*. compared the difference between rat and human intestinal microbiomes and found that the number of observed species in a rat fecal sample was two to three times higher than in two human fecal samples at the same sequencing effort^32^. A similar phenomenon was also captured in our study when we compared the observed and estimated species richness of colonic mucosal samples from rats (1,563 ± 62 for observed and 3,072 ± 219 for estimated richness, respectively) and human beings^14^ (567 ± 84, 1,246 ± 182) (Supplemental Fig. S2).

### Spatial organization of the microbial consortium

The 16S rRNA is coded by the ribosomal RNA operon (*rrn*), which is frequently found in multiple copies. Current analysis pipelines usually produce estimates of per-taxon relative abundances based on the number of 16S genes recovered in a sequence library. Given the variable per-genome copy number of the 16S gene, these pipelines may introduce systematic bias when investigating the genuine community structure of microbiota^33^. To address this issue, the ribosomal RNA operon copy number database (rrnDB)^34^ was incorporated into a copy number-correctional pipeline to describe the microbial consortium at different taxonomic levels along the longitudinal and transverse axes of the rat digestive tract.

**Figure 2:**
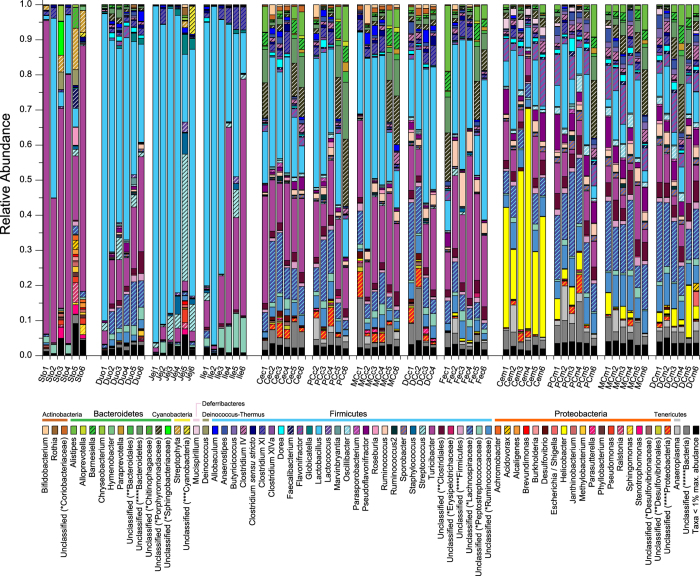
Longitudinal and transverse distribution of dominant taxonomic groups at the genus level. The relative abundance of different genera was adjusted by the 16S rRNA copy number data. Sample ID abbreviations are in line with those used in Fig. 1. The number following the abbreviations stands for the subject number. For example, Sto1, Sto2, Sto3, Sto4, Sto5, and Sto6 stand for the gastric contents from the 1^st^, 2^nd^, 3^rd^, 4^th^, 5^th^ and 6^th^ rat. Unclassified genera under a higher rank are marked by asterisks (*family; **order; ***class; ****phylum; *****kingdom). A complete list of adjusted counts for each genus grouped by sample is presented in supplemental Table S3.

At the phylum level, 21 different taxonomic groups were identified in this study (Supplemental Fig. S3 and Table S3). The majority of taxa belonged to Firmicutes (78.77 ± 2.03%), with the rest distributed amongst Bacteroidetes (9.12 ± 1.56%), Proteobacteria (6.61 ± 1.31%), unclassified bacteria (2.67 ± 0.34%), Tenericutes (0.98 ± 0.21%), Actinobacteria (0.85 ± 0.14%), Cyanobacteria (0.48 ± 0.23%), Deferribacteres (0.30 ± 0.08%) and Deinococcus-Thermus (0.13 ± 0.09%). Other phyla such as *Candidatus* Saccharibacteria (i.e. candidate division TM7), Verrucomicrobia, Elusimicrobia, Lentisphaerae, Euryarchaeota, Acidobacteria, Planctomycetes, unclassified root, Chloroflexi, SR1, WPS-2 and Spirochaetes together comprised, on average, less than 0.1% of total consortium composition. However, only Firmicutes, Bacteroidetes, Proteobacteria and Actinobacteria were found in all samples. Although stomach and duodenum samples showed lower richness and diversity (Fig. 1), they harbored most of these phyla and groups (15 and 16 out of 21), which indicated a high level of phylum diversification within the stomach and duodenum, even though a large portion of phyla were dominated by only a few genera. Chloroflexi was only found in the stomach. The high level of phylum diversification and low level of genus diversification within these phyla could be construed as the result of transient DNA contamination from some environmental microorganisms, which died along the gastrointestinal transit. A similar degree of diversification was also observed in large-intestinal mucosal sites, especially in the distal colonic mucus, which was inhabited by 16 phyla, including the site-specific Spirochaetes. The relative abundances of 7 of the 8 most dominant phyla showed significant differences amongst sampling sites (Supplemental Table S4). Deinococcus, which was also detected in human gastric mucosa^35^, was more abundant in the stomach than other sites. Firmicutes comprised the majority of the microbial population in the small intestine, especially in the ileum (99.34 ± 0.09%). Relative abundances of Actinobacteria and Bacteroidetes were markedly higher in large-intestinal lumen than other biogeographic locations, while Deferribacteres was more abundant in the large-intestinal mucus layer. The relative abundance of Proteobacteria was highest in the cecal mucus and stomach, while the dominant genera within this phylum were completely different in these sampling sites (Fig. 2 and Supplemental Table S4). The relative abundance of Tenericutes was highest in proximal colonic mucus and contents, especially for subject 2. Moreover, Cyanobacteria, which was previously reported to be enriched in a human duodenal mucosal sample^13^, was more abundant in the rat stomach and jejunum but did not reach significance because of large inter-subject variations (FDR = 0.052, Kruskal-Wallis test).

At the genus level, 479 different taxonomic groups were identified (Supplemental Table S3). The relative abundances of 130 genera or groups showed significant differences amongst sampling sites (Supplemental Table S4). In agreement with previous reports^20^,^32^,^36^, Lactobacillus and Turicibacter (27.77 ± 2.91% and 17.04 ± 2.09%, both belonging to Firmicutes) predominated in the rat digestive tract. However, other dominant phylotypes were apt to aggregate in certain anatomic regions. For example, Firmicutes subgroups Lachnospiraceae (genus unclassified, 7.75 ± 0.76%) and Ruminococcaceae (genus unclassified, 3.43 ± 0.31%) were mainly enriched in the colonic mucus. The relative abundance of Helicobacter (3.74 ± 1.23%, belonging to Proteobacteria) was remarkably higher in the cecal mucus layer than other sites^37^. Bacteroidetes subgroup Prophyromonadaceae (genus unclassified, 2.98 ± 0.53%) was abundant in the large-intestinal lumen and mucus (Fig. 2).

For the Lactobacillus genus, the populations from different anatomic sites showed homogeneous compositions, including members from different species, such as *L. hamsteri, L. intestinalis, L. acetotolerans, L. amylolyticus, L. hominis, L. johnsonii, L. taiwanensis, L. gasseri, L. saniviri, L. reuteri, L. antri, L. frumenti, L. pontis, L. mucosae, L. plantarum, L. oeni* and *L. murinus* (Supplemental Fig. S4), as predicted by 16S rRNA sequence alignments and phylogenetic tree predictions. The clade near *L. hominis, L. johnsonii, L. taiwanensis* and *L. gasseri* consisted of the 7 most abundant OTUs, making it the largest OTU clade within the phylogenetic tree and presumably indicating the predominance of these species along the length of rat digestive tract.

Furthermore, inter-subject variations were observed from the phylum to genus levels. Higher inter-subject variations were detected in samples from the upper GI tract than from the lower GI tract (Supplemental Table S5). Network-based analyses further confirmed this trend (Supplemental Fig. S5).

### Stratification of the microbial community

**Figure 3:**
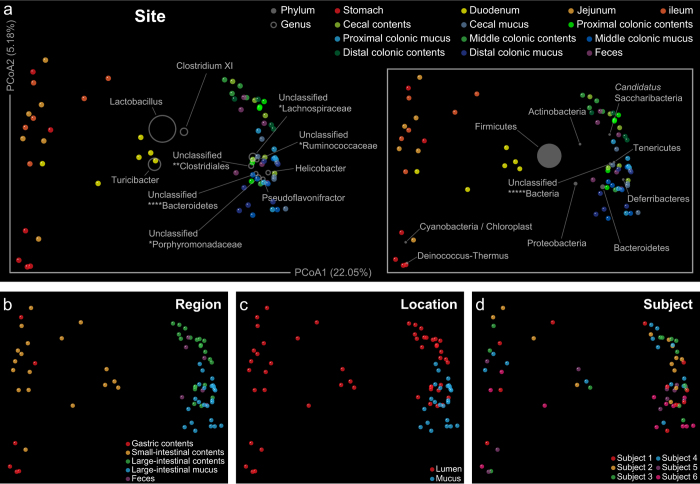
Stratification of the microbial community membership driven by different categorical factors and taxonomic groups. Principal coordinates analysis of unweighted UniFrac distances between samples from rat digestive tract (performed on all OTUs), grouped by sampling (**a**) site (adonis: R^2^ = 0.37; P ≤ 0.001), (**b**) region (adonis: R^2^ = 0.28; P ≤ 0.001), (**c**) location (adonis: R^2^ = 0.08; P ≤ 0.001) and (**d**) subject (adonis: R^2^ = 0.09; P = 0.02). The percent of variation explained by each coordinate is indicated in parentheses. The contribution of different taxonomic groups is represented by the size of the circles (grey) overlaid onto the PCoA based on phylogenetic information. Unclassified genera under a higher rank are marked as in Fig. 2.

The differences in microbial communities between samples, with respect to community membership and structure, were measured by unweighted and weighted UniFrac distances, taking the microbial phylogenetic information into consideration. Community membership, or the presence and absence of microbial lineages, was stratified by different categorical factors, such as anatomic region (adonis: R^2^ = 0.28; P ≤ 0.001), sub-site (adonis: R^2^ = 0.37; P ≤ 0.001), and niche location (adonis: R^2^ = 0.08; P ≤ 0.001). This was further supported by the principal coordinates analysis (PCoA) of unweighted UniFrac distances (Fig. 3a–c), where samples clustered first by major anatomic region then by sub-site, gut regions parsing out based on principal coordinate 1 (22.05% variation explained) and niche location (blocked within the large-intestinal regions) parsing out based on principal coordinate 2 (5.18% variation explained). A similar trend was also detected in community structure taking relative abundances of taxa into account (Supplemental Fig. S6). The taxonomic information overlaid onto the ordination plot illustrates the contribution of each taxonomic group toward sample similarity. PCoA plots with taxonomic information at the phylum and genus levels revealed that the Deinococcus-Thermus and Cyanobacteria were important in clustering of gastric and small-intestinal samples. Meanwhile, Bacteroidetes, Prophyromonadaceae (genus unclassified), *Candidatus* Saccharibacteria contributed to the community similarity of fecal and large intestinal luminal samples, while Proteobacteria, Helicobacter, Deferribacteres, Tenericutes, Clostridiales (family unclassified), Lachnospiraceae (genus unclassified), Ruminococcaceae (genus unclassified) and Pseudoflavonifractor contributed to the community similarity of large-intestinal mucosal samples. In addition, intra-group variability was tested for equal dispersions based on the UniFrac distances, indicating significantly higher variation within the stomach and small intestine than other sites in respect to community membership (Supplemental Fig. S7).

**Figure 4:**
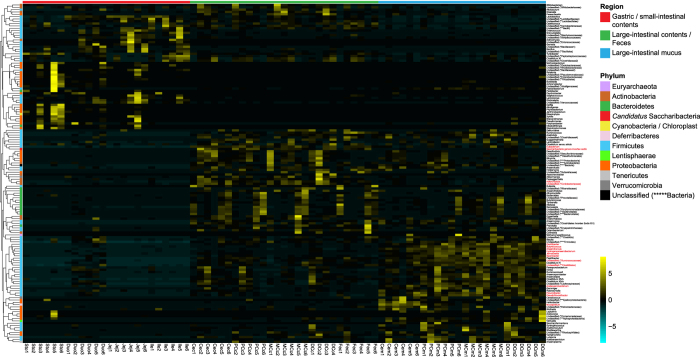
Abundance distribution of genera identified as discriminative variables. To show the distribution of the genera with lower abundance, data were centered and scaled in the row direction. Branches of the tree show taxon similarities. The biogeographic location for each sample and the phylum affiliation for each genus are indicated by different colorations. Genera with VIP > 2 are highlighted in red. Brighter colors along the cyan/black/yellow bidirectional spectrum indicate lower or higher abundances. Unclassified genera under a higher rank are marked as in Fig. 2.

The microbial community relationships between samples were calculated with UPGMA (Unweighted Pair Group Method with Arithmetic Mean) hierarchical clustering. In accordance with previous PCoA results, thirteen sampling sites formed 3 distinct clusters in respect to both membership and structure (Supplemental Fig. S8). To identify key phylotypes responsible for this differentiation, a partial least square discriminate analysis (PLS-DA) was performed based on taxa abundance data at the genus level. The R^2^Y of this model was 0.95, showing high performance for the differentiation. The Q^2^ of this model was 0.82, showing good performance for the prediction. Parameter VIP (variable importance in projection) was used to select genera with the most significant contribution in discriminating the differentiation. A total of 146 genera with VIP > 1 were identified as discriminative variables: 49 genera were enriched in the stomach and small intestine; 50 genera were enriched in the large-intestinal contents and feces; and 47 genera were enriched in the large-intestinal mucus layer. These genera formed 3 distinct blocks on the heat map (Fig. 4), where Adlercreutzia (VIP = 2.37), Coriobacteriaceae (genus unclassified, 2.41), Eubacterium (2.26) and Saccharibacteria (genera *incertae sedis*, 2.28) were the most discriminative taxa in block 2. However, in block 3, Acetanaerobacterium (2.10, proteolytic), Anaerotruncus (2.61), Butyricicoccus (2.39), Flavonifractor (2.23), Hydrogenoanaerobacterium (2.28, proteolytic), Johnsonella (2.15), Pseudoflavonifractor (2.22), Sporobacter (2.36), Clostridiales (family unclassified, 2.04), Ruminococcaceae (genus unclassified, 2.29) and Mucispirillum (2.02, catalase-positive) had the most significant contribution in the generation of this model.

Furthermore, the microbial community was also partitioned by individual subject (adonis: R^2^ = 0.09; P = 0.02 for membership and R^2^ = 0.14; P = 0.003 for structure, respectively), where sample points in PCoA plots were separated by different principal coordinates (Fig. 3d, Supplemental Figs S6 and S9).

### Core microbiota

**Figure 5:**
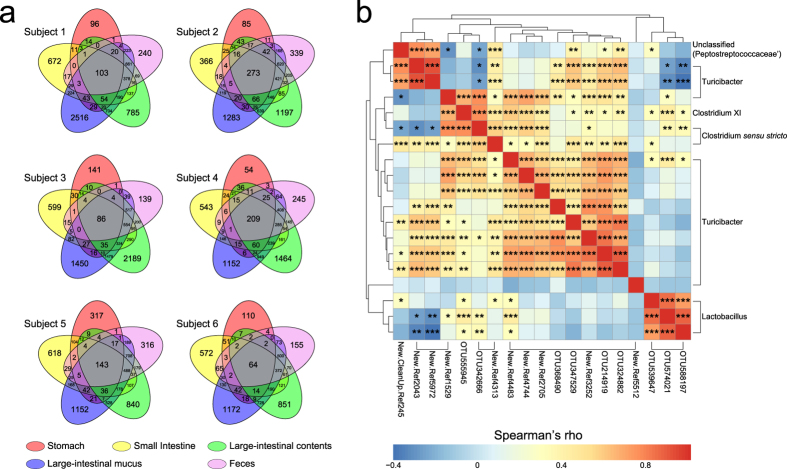
OTU overlap across sampling regions. (**a**) Venn diagrams demonstrating 97% OTU cluster overlap within subjects. Numbers correspond to unique OTU clusters within a subset. To highlight shared OTUs, singleton clusters were removed before analysis. (**b**) Correlation matrix showing the Spearman’s rank correlation amongst the 19 core OTUs shared across all subjects. The correlations were controlled for multiple testing by false discovery rate (*FDR < 0.05, **FDR < 0.01 and ***FDR < 0.001).

For each individual animal, the within-subject overlap of OTU clusters across anatomic regions was calculated with singleton sequences removed. The resulting Venn diagrams demonstrated almost consistent overlap patterns for each subject (Fig. 5a). Large-intestinal contents and mucus had the largest OTU overlap within a subject. Both regions also shared a large number of OTUs with feces, while luminal contents had more overlap than mucus layer except for subject 1. Gastric contents typically shared the fewest OTU clusters with other sampling regions. Interestingly, small-intestinal contents were found sharing slightly more OTUs with large-intestinal mucus than contents in half of the subjects. A subset of 64–273 OTUs was present in every sampling region of each individual, with 19 of these OTUs being present in all subjects accounting for 30.58% of the total sequences. These core OTUs were classified as Clostridium *sensu stricto*, Clostridium XI, Lactobacillus, Turicibacter and Peptostreptococcaceae (genus unclassified) (Supplemental Table S6). The Spearman’s rank correlation test was employed to identify the co-occurrence patterns amongst the core OTUs (Fig. 5b).

Twelve of the 19 core OTU clusters were classified as Turicibacter and were positively correlated with most of the other core OTUs, with the exception of Lactobacillus OTUs. This was exemplified by the lack of correlation between OTU5972 (new reference, Turicibacter) and OTU588197 (greengenes reference, L. reuteri, L. antri and L. frumenti) (rho = −0.413, FDR = 6.16E-4). OTUs from the same genus were positively correlated with each other, such as OTU5972 versus OTU2043 (new reference, Turicibacter) (rho = 0.904, FDR = 0) and also OTU588197 versus OTU574021 (greengenes reference, *L. hominis, L. johnsonii, L. taiwanensis* and *L. gasseri*) (rho = 0.872, FDR = 0).

**Figure 6:**
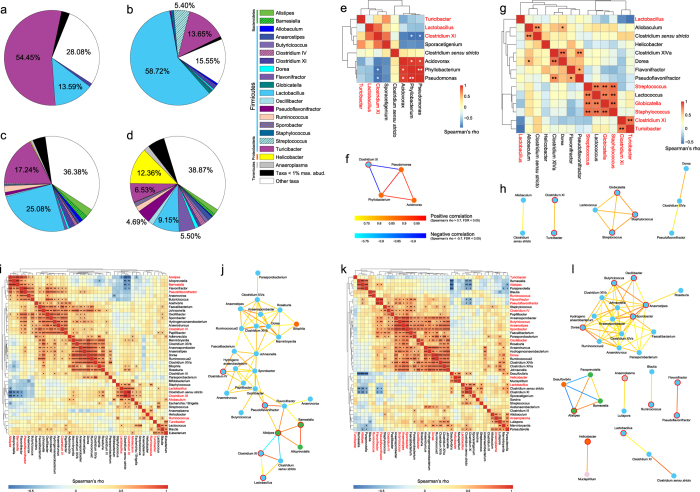
Core microbiota within different GI regions. The composition of core microbiota of (**a**) gastric contents, (**b**) small-intestinal contents, (**c**) large-intestinal lumen and (**d**) mucus layer. The large-intestinal lumen also involves fecal samples, given the fact that colonic contents are precursor of feces. Other taxa refer to non-core genera and unclassified genera under a higher rank. Co-occurrence patterns amongst the core genera across samples from (**e**) gastric contents, (**g**) small-intestinal contents, (**i**) large-intestinal lumen and (**k**) mucus layer, as determined by the Spearman’s rank correlation analysis. The correlations were controlled for multiple testing by false discovery rate and the genera were clustered using the UPGMA hierarchical clustering method (*FDR < 0.05, **FDR < 0.01 and ***FDR < 0.001). Refined co-occurrence networks for (**f**) gastric contents, (**h**) small-intestinal contents, (**j**) large-intestinal lumen and (**l**) mucus layer only based on correlations with absolute value of Spearman’s rho >0.7 and FDR < 0.05. Each node represents a genus, colored according to its phylum-level taxonomic affiliation (Supplemental Fig. S3). Genera with maximum abundance >1% are outlined in red.

For each anatomic region, the core microbiota across subjects was calculated via a detailed phylotype analysis. Different anatomic regions shared different common core microbiota both in amount and composition (Fig. 6a–d). These shared taxa accounted for 71.92, 84.45, 63.62 and 61.13% of the bacterial population in gastric contents, small-intestinal contents, the large-intestinal lumen and the mucus layer, respectively (here the large-intestinal lumen region also involved fecal samples, given the fact that colonic contents are precursor of feces). The stomachs of the 6 rat subjects shared a small set of microbial community composed of 8 bacterial genera (Fig. 6e), with Turicibacter occupying more than half of this core. To identify the co-occurrence patterns amongst these core genera, Spearman’s rank correlation was tested and then a refined network only involving strong correlation with statistical significance (|rho| > 0.7 and FDR < 0.05) was reconstructed. In the co-occurrence network for stomach microbiota (Fig. 6f), three aerobic bacteria belonging to Proteobacteria formed a small robust subnetwork. The presence of aerobes in the stomach may be explained by previous study revealing that the P_O2_ levels in this digestive chamber was about 58 mm Hg for living mice^28^. Furthermore, two of these aerobes Phyllobacterium and Pseudomonas can hydrolyze carbohydrate macromolecules in food, such as starch and insulin.

However, the core microbiota within the small bowel (14 bacterial genera) was found to form a more complex and modular network than that of stomach (Fig. 6g,h). Lactobacillus, which occupied 58.72% of the population in this digestive chamber, only had weak relationships with other genera. On the other hand, four facultative anaerobic genera with relatively low abundance (Globicatella, Lactococcus, Staphylococcus and Streptococcus) composed a small module, which might perform a unique function in this region.

Notably, the large-intestinal lumen (43 genera) and mucus layer (42 genera) had the largest core microbiota (Fig. 6i,k). Despite community memberships overlapping to some extent, the co-occurrence networks for these two adjacent compartments showed distinct topological properties (Fig. 6j,l). In the large-intestinal lumen, core microbiota formed a relatively sparse network (diameter: 8; centralization: 0.18; density: 0.17) where 28 anaerobic organisms belonging to 3 different phyla connected on average to 4.571 neighbors. Most of the members in this network were short-chain fatty acid (SCFA) producers. Interestingly, besides carbohydrate fermentation by saccharolytic bacteria, degradation of plant aromatic compounds might be another source of SCFA in the rat large bowel. Flavonifractor, first isolated from the human intestine, is known to convert quercetin and other flavonoids into acetic and butyric acids^38^. However, flavonoid compounds exist in nature primarily as glycosides, whereas Flavonifractor was previously described to be asaccharolytic^39^. In the co-occurrence network, we found that the neighbors of Flavonifractor, such as Pseudoflavonifractor and Barnesiella, were able to hydrolyze aesculin (similar to flavonoid glycosides in molecular structure) into glucose and aesculetin^38^,^40^. The deglycosylating activity of these bacteria may facilitate the flavonoid conversion by Flavonifractor in the large-intestinal lumen. Meanwhile, bacteria potentially degrading lignocellulose complexes were also found in the network. Sporobacter, Parasporobacterium and Papillibacter were reported to degrade plant polyphenol methyl ethers into acetate and other volatile fatty acids by transferring the methyl group to various sulfides^41^,^42^,^43^. Sulfides may derive from the degradation of sulfur-containing amino acids by proteolytic bacteria, for example, Hydrogenoanaerobacterium^44^ which was closely connected to Sporobacter and Papillibacter in the co-occurrence network. For most of these bacteria, the aromatic ring of plant compounds is cleaved to produce SCFA through the phloroglucinol pathway.

In the large-intestinal mucus layer, the core population of microbes formed several heterogeneous modules. Notably, 15 obligatory anaerobic organisms formed a dense subnetwork (diameter: 3; centralization: 0.43; density: 0.49). Most of the genera in this subnetwork were affiliated to Lachnospiraceae and Ruminococcaceae, with an average of 6.8 neighbors connected to one another. Potentially lignin-degrading bacteria (Parasporobacterium and Sporobacter) also existed in the mucosal co-occurrence network, with their degrees remarkably higher than those in the luminal network. On the other hand, Roseburia, whose degree was 6 in lumen compared to 1 in mucus, was previously reported to possess amylolytic activity^45^,^46^ and may play a role in hydrolysis of resistant starch within the luminal syntrophic saccharolytic network where bacteria were more frequently exposed to non-digestible dietary carbohydrates than in mucus layer. It has been shown that carbohydrate utilization of Roseburia varies significantly between the species and strains, but the genes for starch degradation (GH13s) and the ability to utilize starch *in vitro* are well conserved^47^, indicating that this function plays an important role in Roseburia ecophysiology.

In addition, Johnsonella laid in the center of both networks as hub nodes connected by 8 and 10 neighbors respectively. This non-fermentative and non-proteolytic bacterium was previously reported to be associated with oral diseases^48^ and may play an important role in the co-aggregation of organisms residing in the large-intestinal lumen and mucus layer.

### Shifts of microbial metabolites

Ecological community and environment interact as both cause and effect. After stratification of the microbial community, distinct distribution patterns of microbial metabolites were found along the longitudinal axis of the rat digestive tract. The pH of various anatomic sites also differed, with the gastric contents having a relatively low pH (5.01 ± 0.29) and the rest being near neutral (Supplemental Table S7). The stomach was traditionally believed to represent a harsh environment for microorganisms due to low-pH acidic conditions. However, in our study the gastric digesta was found to be only moderately acidic, which is in agreement with earlier research indicating that gastric pH could increase with ingestion of meals^49^. Measurements of microbial metabolites revealed that the microbial community in the large intestine was more active than in the stomach and small intestine, with the cecum being the most active fermentation chamber. Lactate was the most predominant SCFA (accounting for more than 80%) in the stomach and small intestine (Supplemental Fig. S10), which may be linked with the predominance of Turicibacter, Lactobacillus and Streptococcus in these regions. These organisms were characterized as producing lactate as their main fermentative end-product^50^,^51^.

**Figure 7:**
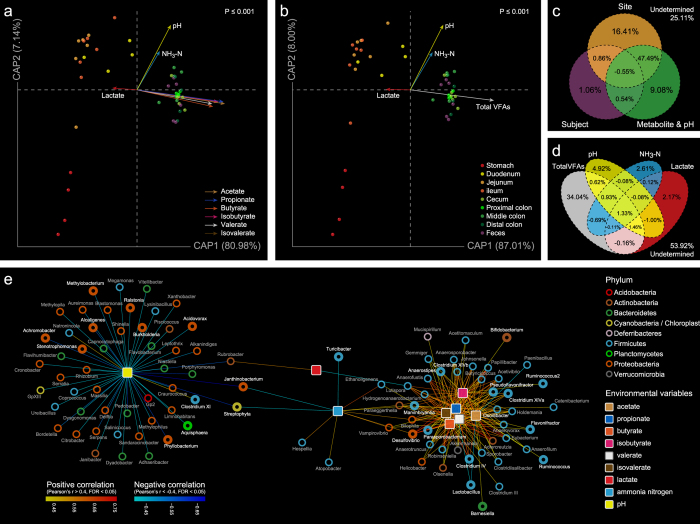
Correlations between environmental variables and microbial community membership. Constrained analysis of principal coordinates of unweighted UniFrac distances and environmental variables for luminal samples. Arrows indicate the direction and magnitude of environmental variables associated with bacterial community membership. The P-value of the Monte Carlo permutation test is shown on the upper left. (**a**) Each type of VFA was plotted independently. (**b**) The concentrations of several VFAs were summed up and plotted as total VFAs. Venn diagrams demonstrating percentages of community membership variation explained by (**c**) biogeographic location, inter-individual variability, environmental variable and separately explained by (**d**) different environmental variables (pH, total VFAs, lactate and ammonia nitrogen). (**e**) Correlation network of environmental variables and taxonomic groups at the genus level with absolute value of Pearson’s r >0.4 and FDR < 0.05. Genera with maximum abundance >1% are highlighted in bold.

Constrained analysis of principal coordinates (CAP) was performed to discern the possible relationships between the microbial community and different environmental variables. In this case, instead of correlating the environmental variables a *posteriori* with the main ordination axes of the analyzed data set, we constrained the ordination axes themselves to be linear combinations of the supplied set of environmental variables. The length of a variable arrow indicating the gradient direction is proportional to the strength of the relationship of that variable to community composition. In the biplot of unweighted UniFrac CAP, samples from different anatomic regions were mainly separated by axis 1 (80.98% constrained variation explained) to which various volatile fatty acids (VFAs) and lactate were positively and negatively related, respectively (Fig. 7a). Various VFAs showed similar gradient directions where acetate and propionate appeared to be the most important environmental variables (Mantel: r = 0.39; P ≤ 0.001 for acetate and r = 0.41; P ≤ 0.001 for propionate, respectively). When the concentrations of various VFAs were summed up, the above trend remained evident (Fig. 7b), with total VFAs representing the greatest correlation with the community membership (Mantel: r = 0.35; P ≤ 0.001).

Variance partitioning analysis (VPA) was further performed to assess the contributions of different categorical factors and environmental variables to the microbial community variance. The results of unweighted UniFrac VPA showed that 74.89% of the variance could be explained by these constraints. The biogeographic location, inter-individual variability and environmental variable could independently explain 16.41, 1.06, and 9.08% of the variation of microbial community membership, respectively. However, 47.49% of the variation was co-explained by biogeographic location and environmental variable, meaning that the community membership and the environmental data had a fairly similar spatial structuring in the rat gut (Fig. 7c). Amongst these environmental variables, total VFAs could independently explain 34.04% of the variation, followed by pH (4.92%), ammonia nitrogen (NH_3_-N, 2.61%) and lactate (2.17%, Fig. 7d). Note that in the variance partitioning theory, the percentage value of co-contribution for multiple variables can be negative^52^ although in ecological practice, however, this is unlikely to occur. A similar trend was also observed by weighted UniFrac CAP and VPA for the community structure (Supplemental Fig. S11), apart from less overlap in contributions for biogeographic location and environmental variable (21.72%).

To further explore the responses of particular community members to the gradients of environmental variables across the biogeographic locations, the correlations between different environmental variables and taxonomic groups at the genus level were tested (Supplemental Fig. S12), then a network was constructed, where two topologically distinct modules respectively centered by different VFAs and pH were connected through lactate and NH_3_-N as bridges (Fig. 7e). Various VFAs co-aggregated together (average degree: 31.67; neighborhood connectivity: 5.47; topological coefficient: 0.64) surrounded by taxa mainly belonging to Firmicutes, which may imply a better adaptability of these bacteria to the ecological circumstances where complex anaerobic fermentation provides an energetic advantage^53^ and a possible signaling communication strategy amongst these organisms. It has been noticed that in complex microbial ecosystems, bacterial metabolites may act as chemical signal substances detected by a species to monitor the abundance of itself or its neighbors, which is known as quorum sensing^54^. On the other hand, most of the Proteobacteria and Bacteroidetes phylotypes were negatively correlated to pH (degree: 52; neighborhood connectivity: 1.06; topological coefficient: 0.03), forming an asterisk-like structure.

### Differences amongst murine gastrointestinal microbiota

**Figure 8:**
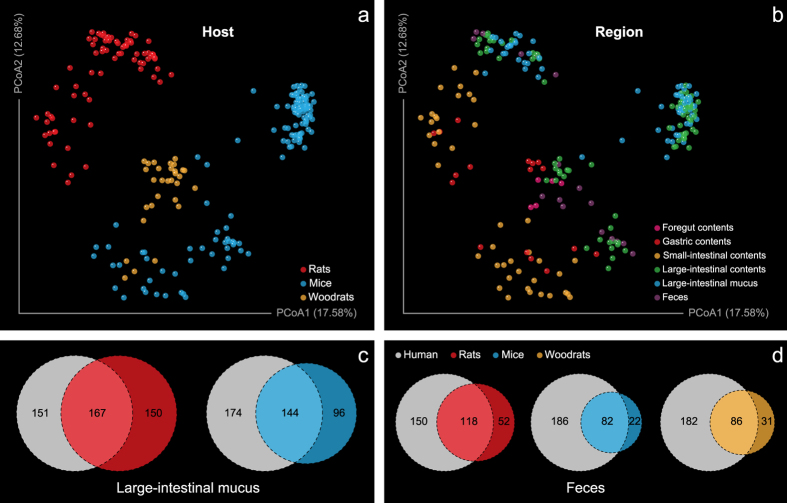
Differences in microbial biogeographies of rodent and human digestive tract. Principal coordinates analysis of Bray-Curtis distances between samples from mouse, rat and woodrat digestive tract (performed on all taxonomic groups at the genus level), grouped by (**a**) host (adonis: R^2^ = 0.25; P ≤ 0.001) and (**b**) anatomic region (adonis: R^2^ = 0.17; P ≤ 0.001). The percent of variation explained by each coordinate is indicated in parentheses. Venn diagrams demonstrating the taxon overlap between different hosts at the genus level for (**c**) large-intestinal mucus and (**d**) feces.

Historically, laboratory rodents have significantly contributed to the knowledge in several areas of human health and disease. To investigate the biological significance of the biogeographic map of rats, we compared the gastrointestinal microbiota from different murine animals. In different amplicon sequencing projects, researchers chose different protocols targeting various hypervariable regions in the 16S rRNA gene, which could cause difficulties to OTU binning for lacking alignable sequences. Here, a phylotype-based strategy was employed, where sequences of different variable regions were processed through an identical pipeline to obtain comparable phylotype abundance data. The ordination result for rat GI microbiota based on this strategy showed great similarities with those of Unifrac PCoA (Supplemental Fig. S13, protest: r = 0.95; P ≤ 0.001 for unweighted and r = 0.81; P ≤ 0.001 for weighted). The ordination result for mouse, rat and woodrat gastrointestinal microbiota showed that the rat microbial biogeography represented a remarkably distinct landscape, where sample points mainly clustered according to the metadata recording host information (Fig. 8a). Besides the host genotype (adonis: R^2^ = 0.25; P ≤ 0.001), anatomic region was another driver (adonis: R^2^ = 0.17; P ≤ 0.001) for the stratification of murine gastrointestinal microbiota (Fig. 8b). The sample similarity within a given anatomical region predominated in the PCoA plot in spite of the strong contribution of host genotype background variability. The dispersion pattern of intra-region variability was consistent with that found in rats when taking these murine gastrointestinal microbiota together into consideration (Supplemental Figs S7 and S14). Despite the difference in the relative abundance (Supplemental Fig. S15), in the view of phylotype overlap, of the 3 rodents, the gastrointestinal microbiota of rats was most closely related to that of human, especially in the large-intestinal mucus layer (Fig. 8c,d). Nevertheless, these results must be interpreted with caution, because these amplicon sequences were derived from different studies using different DNA extraction methods, polymerase chain reaction (PCR) amplification protocols and sequencing platforms.

## Discussion

The rat is a long-standing animal model in biomedical research. More than a million publications on studies in which rats were used have been reported, illustrating the substantial amount of data that has been gathered on this species^55^. The first rat genomic sequence was published in 2004^56^ and was the third mammalian genome to be sequenced after those of the human and the mouse. However, the intestinal microbiome, as the second genome for rats, has not been well characterized. Our knowledge about the spatial structure of the microbial consortium in normal rats is still very limited. Therefore, an investigation into the radial microbiota diversity and composition along the length of the rat digestive tract was carried out in the present study using a high-throughput 16S rRNA gene sequencing approach.

The sequencing results showed that the microbial community diversity increased along the longitudinal axis of the rat digestive tract, which was in conflict with the tendency previously found in mice, where gastric and duodenal samples had similar levels of diversity with large-intestinal samples. Gu *et al*. hypothesized that the existence and “vanishing” of “transient microbiota” could explain the relatively high diversity in the stomach and duodenum^57^. Nevertheless, in our results the absolute SI values were close to those in mice for these anatomic sites, whereas in the large intestine the diversity measurements were much higher, especially for the colonic mucus layer. This presumably implies that the rat’s large intestine houses a more complex micro-ecosystem, which was also supported by early research indicating that the number of bacterial species in rat feces was 2–3 times higher than that of human feces at the same sequencing effort^32^. A similar phenomenon was also captured in our study when comparing the colonic mucosal samples from rats and human beings.

Given the variable per-genome copy number of the 16S gene in bacteria and archaea, a copy number-corrected pipeline was employed to describe the microbial community composition in the rat GI tract. Besides Lactobacillus, a common commensal organism usually highly abundant in the laboratory rodent gut, another lactate-producing bacterium, Turicibacter, was also predominant in the rat digestive tract. The enrichment of this microorganism throughout the alimentary canal, especially in the stomach, might be a trait of rats and not of other murine animals. However, there are very few reports describing Turicibacter species and their biological and clinical significance. *Turicibacter sanguinis*, the only species systematically described within this genus, was isolated from blood culture of a febrile patient with acute appendicitis^50^. Turicibacter rRNA gene sequences have also been PCR-amplified from human feces^58^ and ileal pelvic pouch contents from a subject with ulcerative colitis^59^. Presley *et al*. examined the bacteria-immune system interactions in mice and proposed a potential association between Turicibacter and immunoregulatory cells^60^. To date, there are only two published whole-genome sequencing projects for this genus^61^,^62^ and more cumulative work is further required to explore the potential interaction between this microorganism and its host.

The relatively high abundance of lactate-producing bacteria in the upper GI tract probably explains the enrichment of lactate (accounting for more than 80% of SCFA) in the stomach and small intestine. Recent evidence for diverse and transcriptionally active microbiota within the stomach has challenged the traditional notion that the upper GI tract is nearly sterile^35^,^63^,^64^. However, the metabolic activity of Turicibacter, previously described as strictly anaerobic, needs to be further studied given the oxygen availability in the upper GI tract. On the other hand, the fermentative pattern in the lower GI tract was totally different from the upper, where increasing productions of various VFA were observed, especially for butyrate, a preferred energy source for colonic epithelial cells^65^. Detailed phylotype analysis revealed that the VFA producers Lachnospiraceae and Ruminococcaceae constituted the bulk of the core microbiota in the large intestine. Interestingly, besides carbohydrate fermentation, degradation of plant aromatic compounds might be another source of VFAs in the rat large bowel. In the microbial co-occurrence network, several potential function modules were identified where bacteria cooperate with each other to convert flavonoid and lignin complexes into acetate and butyrate. Similar to the so-called quorum sensing mechanism^54^, there may exist a communication strategy amongst these ecosystem service providers using their metabolites as chemical signaling^53^.

VPA for rats showed that the GI microbiota community variation could be explained mostly by the biogeographic location and environmental variable. Unlike human beings, where inter-individual variability is a hallmark of GI microbiota^14^, the inter-individual variability of rats only contributed to a small part of the variation. This is not surprising because the animals used here have similar genetic composition and diet condition, in addition to the coprophagy of rats. Nevertheless, different anatomic regions in the digestive tract have distinct niche-specific physicochemical conditions, such as intestinal flow rate, redox potential, oxygen concentration, availability of nutrients and host immune response^26^,^27^,^29^. In accordance with previously imaged oxygen gradient^28^, the core microbiota in rat evolved from aerobic to facultative anaerobic to obligatory anaerobic along the longitudinal axis. The community membership and structure were mainly stratified by biogeographic factors such as anatomic region and sub-site, whereas the intra-region variability was significantly higher within the stomach and small intestine than other segments. This trend was still evident when comparing different murine gastrointestinal microbiota, even if host genotype background was also a strong driver. The ordination result showed that the rat GI microbial biogeography characterized in present study might provide a new reference for digestive tract-related disease research and that the murine animals may have co-evolved with their gut microbiota^30^.

Despite having the largest number of overlapping OTUs, the luminal and mucosal microbial communities showed notably radial segregation in the rat lower GI tract. PLS-DA for these two adjacent compartments indicated that the key phylotypes responsible for this differentiation included several mucus-enriched proteolytic and catalase-positive microorganisms, which is in agreement with an earlier report^66^. The saccharolytic bacteria residing in the outer mucus layer may digest mucin glycans^67^ and help make polypeptides more accessible for proteolytic bacteria, enhancing syntrophic interactions. The topologically distinct co-occurrence networks of core microbiota for these intimate regions may fulfill different roles within this spatially defined ecosystem.

In conclusion, we have generated the first comprehensive characterization of the normal rat microbiota landscape, which will improve our understanding of the mechanism within various GI niches of this long-standing murine model as well as the development of dysbiotic microbiota associated with diseases. The rat digestive tract harbors many distinct niches, each containing a different microbial ecosystem that might provide indispensable services to the host. Our study also confirms that fecal samples cannot represent the whole microbiota in the digestive tract, and attention should therefore be paid to ensure that the proper GI samples are selected and used for gut-related disease research.

## Methods

### Chemicals and Reagents

Analytical standards (acetic, propionic, isobutyric, butyric, isovaleric and valeric acids, lithium L-lactate, Ammonium sulfate) and sodium nitroprusside were purchased from Sigma-Aldrich Company (St. Louis, MO, USA). P-hydroxydiphenyl was purchased from J&K Scientific Ltd. (Beijing, China).

### Animals and sample collection

Six male-specific pathogen-free (SPF) Sprague-Dawley rats aged 6 weeks (Shanghai Laboratory Animal Center, Shanghai, China) were used in this study.

All of the protocols used in this study were approved by the Ethics Committee of Jiangnan University, China (JN NO. 20140926-0204-35-1), and the procedures were carried out in accordance with the European Community guidelines (Directive 2010/63/EU) for the care and use of experimental animals. All rats were co-housed in one cage in a temperature- and humidity-controlled room under a strict 12 hour light–dark cycle. Irradiation sterilized commercial rodent chow (main ingredients: corn, soybean meal, wheat and fish meal. Shuangshi animal feed Technology Ltd. Suzhou, Jiangsu, China) and autoclaved water were given *ad libitum* during the one-week acclimatization period, after which feces were collected. Rats were transferred to separate sterilized cages, and feces were collected in individual clean EP tubes (Axygen Scientific, Union City, CA, USA). After this, the rats were weighted and then euthanized by intraperitoneal injection of 200 mg/kg sodium pentobarbital euthanasia solution. Abdomens were rinsed in 75% ethanol and dried with a paper towel before incision. Stomach, duodenum, jejunum, ileum, cecum and colon were excised and immediately weighed as a whole. The colon was subsequently divided equally into three parts as proximal, middle, and distal. Each GI section was then placed in a clean petri dish and cut lengthwise. The luminal contents were carefully emptied into the petri dish using forceps without scraping the surface and then collected into clean EP tubes. The gastrointestinal wall was then washed twice with phosphate-buffered saline, and the mucus layer was scraped off with a rubber spatula then stored at −80 °C for further microbiota analysis. Luminal contents from each GI section and feces were divided into aliquots for various uses (described below) and stored at −80 °C until analysis.

### Determination of microbial metabolites

A portion of luminal samples were divided into aliquots for the determination of pH, volatile fatty acids (VFAs), lactic acid and ammonia nitrogen.

To determine the pH, samples (50 mg) were diluted 1:5 in Milli-Q water. The pH was measured using a Mettler Toledo pH meter with an InLab^®^ Solids pH electrode (Mettler Toledo, Columbus, OH, USA).

To determine VFAs, samples (50 mg) were resuspended in 500 μL of saturated NaCl solution and acidified with 20 μL of sulfuric acid (10%); 800 μL of diethyl ether was then added to extract the fatty acids. After shaking for 2 min, the mixture was centrifuged at 14,000 g for 15 min. The supernatant was then dried using 0.25 g of Na_2_SO_4_ and the concentration of VFAs (acetic, propionic, isobutyric, butyric, isovaleric and valeric acids) was analyzed via Gas Chromatography (GC-2010 plus, Shimadzu, Kyoto, Japan) fitted with a quadrupole Mass Spectrometry unit (GCMS-QP2010 Ultra, Shimadzu, Kyoto, Japan), which was equipped with a Rtx-Wax column (length = 30 m, id = 0.25 mm, thickness = 0.25 μm, Restek, Evry, France). Helium was used as carrier gas at a constant linear velocity of 35.0 cm/sec. The injection volume was 1 μL with a split ratio of 1:10. The injection temperature was set at 240 °C and the GC temperature program was as follows: begin at 100 °C, increase to 140 °C at the rate of 7.5 °C/min, followed by 60 °C/min to 200 °C, and hold at 200 °C for 3 min. The ion source temperature was set at 220 °C. The analytes were detected using the single scan mode (acetic, butyric, isovaleric and valeric acids: 60 m/z; propionic acid: 57 m/z; isobutyric acid: 43 m/z). The concentration of VFAs was calculated using the external standard method and expressed as μmol/g sample.

Lactic acid in samples (50 mg) was determined by the p-hydroxydiphenyl method as previously described^68^. Lithium L-lactate was used as a standard and the concentration of lactic acid was expressed as μmol/g sample.

Ammonia nitrogen in samples (50 mg) was determined by the indophenol method as previously described^69^. (NH_4_)_2_SO_4_ was used as a standard and the concentration of NH_3_-N was expressed as μmol/g sample.

### DNA extraction, PCR amplification, quantification, and sequencing

Total genomic DNA was extracted from all samples (100 mg) using FastDNA^®^ Spin Kit for soil (MP Biomedicals, Santa Ana, CA, USA) according to the manufacturer’s instructions. DNA quality was assessed by the 260/280-nm and 260/230-nm absorption ratios, measured with an ND-2000 spectrophotometer (Nanodrop Inc., Wilmington, DE, USA) and agarose gel electrophoresis.

Due to the low biomass of several mucus samples, a nested PCR protocol was used to amplify the bacterial 16S rRNA gene from all samples. For the first round of amplification, the near full-length 16S rRNA gene was amplified using primers 27F (5′-AGAGTTTGATCCTGGCTCAG-3′) and 1492R (5′-GGTTACCTTGTTACGACTT-3′). In the second round, modified fusion primers 520F (5′-AYTGGGYDTAAAGNG-3′) and 802R (5′-TACNVGGGTATCTAATCC-3′) containing a seven-base barcode were used to amplify the V4 region. Ten cycles were used for the first PCR round and 30 cycles were used for the second round. The PCR amplification was carried out with the GoldStar Best High-Fidelity DNA polymerase (CWBIO, Beijing, China) following the manuals. The first round of amplification consisted of an initial denaturation step at 95 °C for 10 min, followed by 10 cycles, where 1 cycle consisted of 94 °C for 30 s (denaturation), 55 °C for 30 s (annealing) and 72 °C for 1 min 30 s (extension), and a final extension of 72 °C for 5 min. The procedure for the second round of PCR amplification was 95 °C for 10 min; 94 °C for 30 s, 50 °C for 30 s, and 72 °C for 30 s, repeated for 30 cycles; and 72 °C for 5 min. Negative controls were always performed to verify the lack of amplification without DNA template. PCR products were excised from a 1.5% agarose gel, recovered using QIAquick^®^ Gel Extraction Kit (QIAGEN, Hilden, Germany) and quantitated using the Quant-iT^TM^ PicoGreen^®^ dsDNA Assay Kit (Life Technologies, Grand Island, NY, USA). Libraries were prepared using TruSeq Nano DNA LT Kit (Illumina, catalog no. FC-121-4001) and sequenced with the Miseq Reagent Kit v3 (600 cycles-PE, Illumina, catalog no. MS-102-3001).

### Bioinformatic Analysis

A unique barcode was used to label each sample and multiplex Illumina sequencing resulted in 4,957,802 paired-end reads. Forward and reverse raw sequences were assembled using SeqPrep (https://github.com/jstjohn/SeqPrep) in QIIME package^70^ (Quantitative Insights Into Microbial Ecology, v1.9.1) with default parameters (minimum allowed overlap: 15 bp; minimum allowed fraction of matching bases: 90%; maximum mismatched high quality bases: 2%). Reads that could not be assembled were discarded. Sequences with fraction of consecutive high quality base calls (phred score >29) lower than 75%, containing ambiguous bases, or lacking a perfect match to given barcodes were removed. When a sequence had more than 3 consecutive low quality base calls (phred score <30), it was truncated. Reads identified as host genome were exclude by blast against the rat reference genome^71^ (Ensembl assembly Rnor 6.0, e-value < 1e-10, % alignment >0.97).

Raw reads of 42 samples from 6 C57BL/6 mice^57^, 2 samples from 3 C57BL/6 mice^72^ and 30 samples from 5 woodrats (*Neotoma albigula*)^73^ were downloaded from the NCBI Short Read Archive (SRA) under accession number SRA061180, ERP000288 and SRP022360, respectively. Raw reads of 85 samples from 6 C57BL/6 mice^74^ were downloaded from Figshare under accession number 1499145. Raw reads of 17 samples from 4 human beings^14^ were downloaded from the Rapid Annotation using Subsystems Technology for Metagenomes database (MG-RAST) under accession number mgp1982. These reads were filtered as above and used in further analysis combining the reads from rats. To exclude host genome contamination, reads from mice and human beings were blasted against reference genome (Ensembl assembly GRCm 38.71 and GRCh 38) while reads from woodrats were not screened for lack of host genome data.

An open reference OTU picking pipeline was performed against the greengenes database^75^ (release 13.8) to cluster reads with >97% similarity using UCLUST^76^ v1.2.22. Representative sequences from each cluster were aligned with the PyNAST aligner^77^ to the greengenes core set, and then a *de novo* taxonomic tree was constructed using FastTree^78^ v2.1.3.

The taxonomic abundance of each sample was calculated with Ribosomal Database Project (RDP) classifier^79^ v2.10.2 trained with 16S rRNA training set No. 14 using a bootstrap cutoff of 50%. Subsequently, a gene copy number adjustment for 16S rRNA sequences was performed. The 16S rRNA copy number data is provided by rrnDB website^34^ (https://rrndb.umms.med.umich.edu/). A customized script was used to extract and merge abundance data at different taxonomic levels.

To minimize the difference of sequencing depth amongst samples, a rarefied OTU subset was generated by evenly resampling 22,880 sequences per sample (without replacement) for further alpha and beta diversity analysis. OTU richness, Chao1 richness estimator, Shannon index (SI) phylogenetic diversity (PD) were used to estimate the alpha diversity in each sample, involving ten resampled data (without replacement). The differences amongst the microbial communities were determined based on the phylogenetic information within each sample with both the unweighted and weighted UniFrac analysis^80^. UPGMA (Unweighted Pair Group Method with Arithmetic Mean) trees of these metrics were calculated within QIIME and plotted using FigTree v1.4.2. Jackknife values were calculated with 100 sets of 18,000-sequence rarefied tables of 75 samples and with 100 sets of 16,000-sequence rarefied tables of 13 pooled samples (sequences were merged within anatomic sites across subjects). Principal coordinates analysis (PCoA) and constrained analysis of principal coordinates^81^ (CAP) of unweighted and weighted UniFrac distances were calculated in QIIME and R-package vegan^82^ then visualized by EMPeror^83^.

In order to inspect the composition of lactobacilli to a higher-level resolution, a phylogenetic tree of representative sequences of dominating Lactobacillus OTUs and Lactobacillus-type strains was constructed using MAGE^84^ 6.06, then visualized by Interactive Tree of Life^85^ (iTOL, http://itol.embl.de/). The 16S rRNA sequences of bacteria affiliated to Lactobacillus were retrieved from the GenBank database according to the List of Prokaryotic names with Standing in Nomenclature^86^ (LPSN, http://www.bacterio.net/).

### Statistical Analysis

The differences in alpha diversity measurements, relative abundances of taxa, pH and concentrations of microbial metabolites amongst different groups were analyzed using Kruskal-Wallis rank sum test by stats package in R^87^. Nemenyi’s *post hoc* multiple comparison test between all possible group combinations was performed by kruskalmc implemented in R-package pgirmess. P-values were adjusted for multiple testing by false discovery rate (FDR) using the Benjamini & Hochberg method^88^.

Permutational multivariate analysis of variance^89^ (PERMANOVA) was employed to describe the strength and significance that a categorical factor has in determining variation of ecological distances. Variance partitioning analysis^90^ was performed to assess the contributions of biogeographic location, inter-individual variability and different environmental variables (pH, SCFAs and ammonia nitrogen) to the microbial community variance. The correlations between community membership or structure, as measured by unweighted and weighted UniFrac distances, and different environmental variables, were assessed using Mantel test^91^. The significance of the results from CAP, PERMANOVA and Mantel tests was tested by 999 Monte Carlo permutations. PERMANOVA was performed using adonis implemented in vegan.

Partial least square discriminate analysis (PLS-DA) was performed based on the relative abundances of genera using Simca-p + 11.0 (Umetrics AB, Umea, Sweden). R^2^ was the index of how much the model can explain over all the cases. Q^2^ was the index of how much the model can predict over all the cases. Variable importance in projection (VIP) reflects the discriminative power of variables. Variables with VIP > 1, which were important contributors to generation of the model, were used to build a heat map in R studio using pheatmap package.

Spearman’s rank correlation was used to evaluate the co-occurrence relationships between taxa. Correlations between abundances of particular genera and gradients in pH as well as microbial metabolites were determined using Pearson’s correlation coefficient by rcorr implemented in R-package Hmisc. False discovery rate values were estimated using the Benjamini & Hochberg method to control for multiple testing. Correlation networks were visualized by Cytoscape^92^ v2.8.1 using the force-directed method for laying out the graphs.

In order to compare samples targeting different hypervariable regions in the 16S rRNA gene, filtered sequencing data from different studies were processed with the identical taxa annotation pipeline, then the taxonomic abundance data at the genus level were extracted and combined to calculate Bray-Curtis dissimilarities amongst different samples. To remove sampling depth heterogeneity, abundance data were square-root transformed and Wisconsin double-standardized. A principal coordinates analysis (PCoA) was then performed based on the Bray-Curtis distance matrix. To assess similarities between different community ordinations, a Procrustes analysis was performed within vegan and visualized by EMPeror. Venn diagrams were constructed using R-package VennDiagram.

To test for intragroup dispersion, inter-sample UniFrac and Bray-Curtis distances were tested for equal intra-group dispersions using betadisper^93^ implemented in vegan. The significance was calculated using ANOVA with Tukey’s HSD *post hoc* test.

### Data access

The sequencing data from this study were deposited in the NCBI Sequence Read Archive (SRA) under accession no. SRP076251.

## Additional Information

**How to cite this article:** Li, D. *et al*. Microbial Biogeography and Core Microbiota of the Rat Digestive Tract. *Sci. Rep.* **7**, 45840; doi: 10.1038/srep45840 (2017).

**Publisher's note:** Springer Nature remains neutral with regard to jurisdictional claims in published maps and institutional affiliations.

## Supplementary Material

Supplementary Information

Supplementary Table S3

Supplementary Table S4

Supplementary Table S6

Supplementary Table S7
