# Supplementary material for: Microbial Biogeography and Core Microbiota of the Rat Digestive Tract
Source: Sci Rep. 2017 Apr 4;7:45840. doi: 10.1038/srep45840 (PMC5379200; doi:10.1038/srep45840)
Supplement: Supplementary Information [file srep45840-s1.pdf]

Microbial Biogeography and Core Microbiota of the Rat Digestive Tract

Dongyao Li[1], Haiqin Chen[1]*, Bingyong Mao[1], Qin Yang[1], Jianxin Zhao[1], Zhennan Gu[1], Hao Zhang[1], Yong Q. Chen[1,2], Wei Chen[1,3]*

[1]State Key Laboratory of Food Science and Technology, School of Food Science and Technology, Jiangnan University, Wuxi 214122, P.R. China.

[2]Departments of Cancer Biology and Biochemistry, Wake Forest School of Medicine, Winston-Salem, NC 27157, USA.

[3]Beijing Innovation Centre of Food Nutrition and Human Health, Beijing Technology and Business University (BTBU), Beijing 100048, P.R. China.

*****Corresponding authors: Haiqin Chen. Address: School of Food Science and Technology, Jiangnan University, 1800 Lihu Avenue, Wuxi 214122, P.R. China. Tel: (86) 510-85197239; Fax: (86) 510-85197239; Email: haiqinchen@jiangnan.edu.cn.

Wei Chen. Address: School of Food Science and Technology, Jiangnan University, 1800 Lihu Avenue, Wuxi 214122, P.R. China. Tel: (86) 510-85912155; Fax: (86) 510-85912155; Email: weichen@jiangnan.edu.cn.

Supplementary Figures

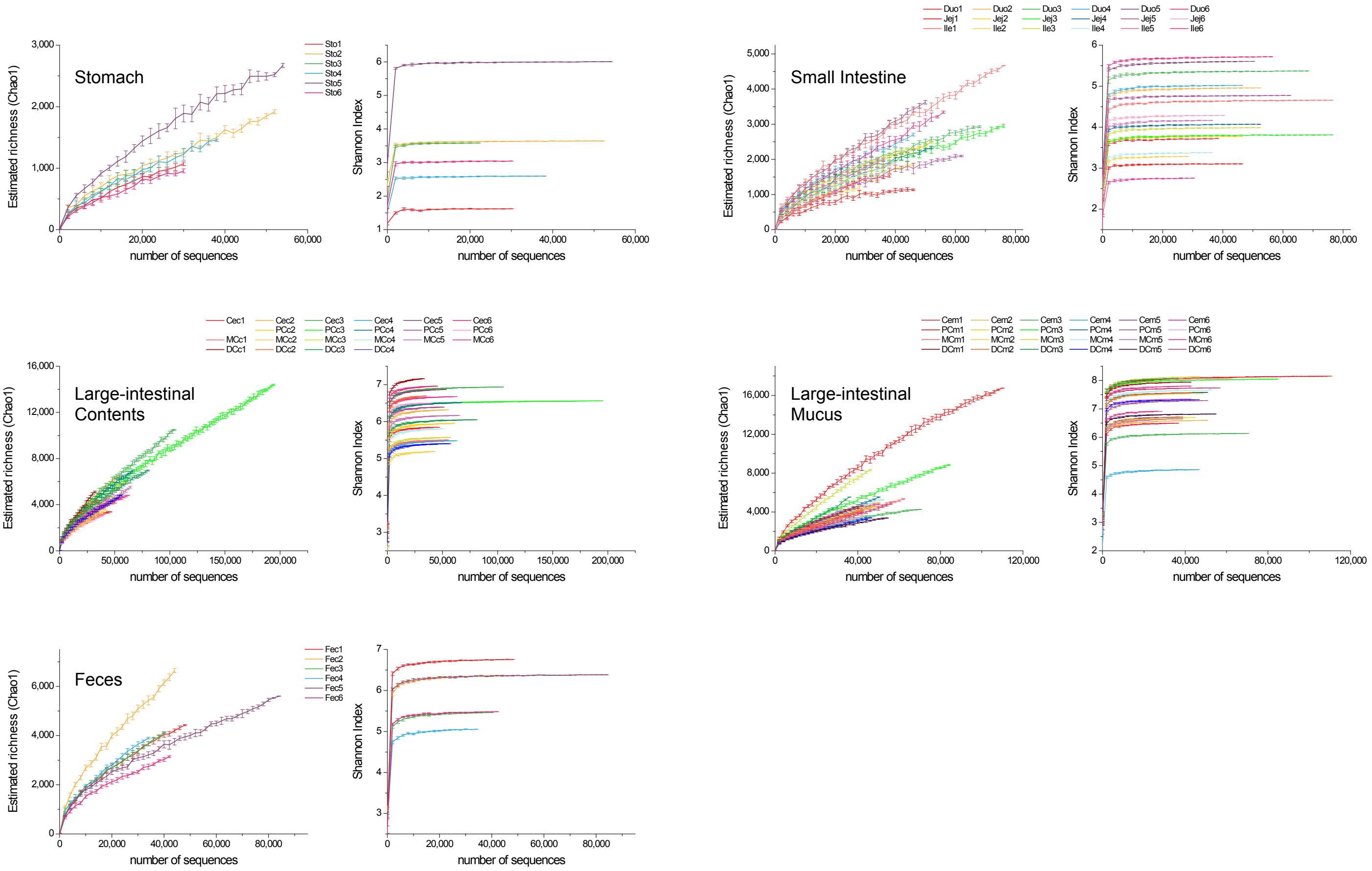

Figure S1. Alpha rarefaction curves. Estimated richness and Shannon index for all samples grouped by region. The average Chao1 estimator and Shannon diversity index (SI) are presented from ten iterations of rarified subsets of all sequences (on the x-axis) and error bars represent the upper and lower limits of the 95% confidence interval. Sto: gastric contents; Duo: duodenal contents; Jej: jejunal contents; Ile: ileal contents; Cec: cecal contents; PCc: proximal colonic contents; MCc: middle colonic contents DCc: distal colonic contents; Fec: feces; Cem: cecal mucus; PCm: proximal colonic mucus; MCm: middle colonic mucus; DCm: distal colonic mucus. The number following the abbreviations stands for the subject number. For example, Sto1, Sto2, Sto3, Sto4, Sto5, and Sto6 stand for the gastric contents from the 1st, 2nd, 3rd, 4th, 5th and 6th rat.

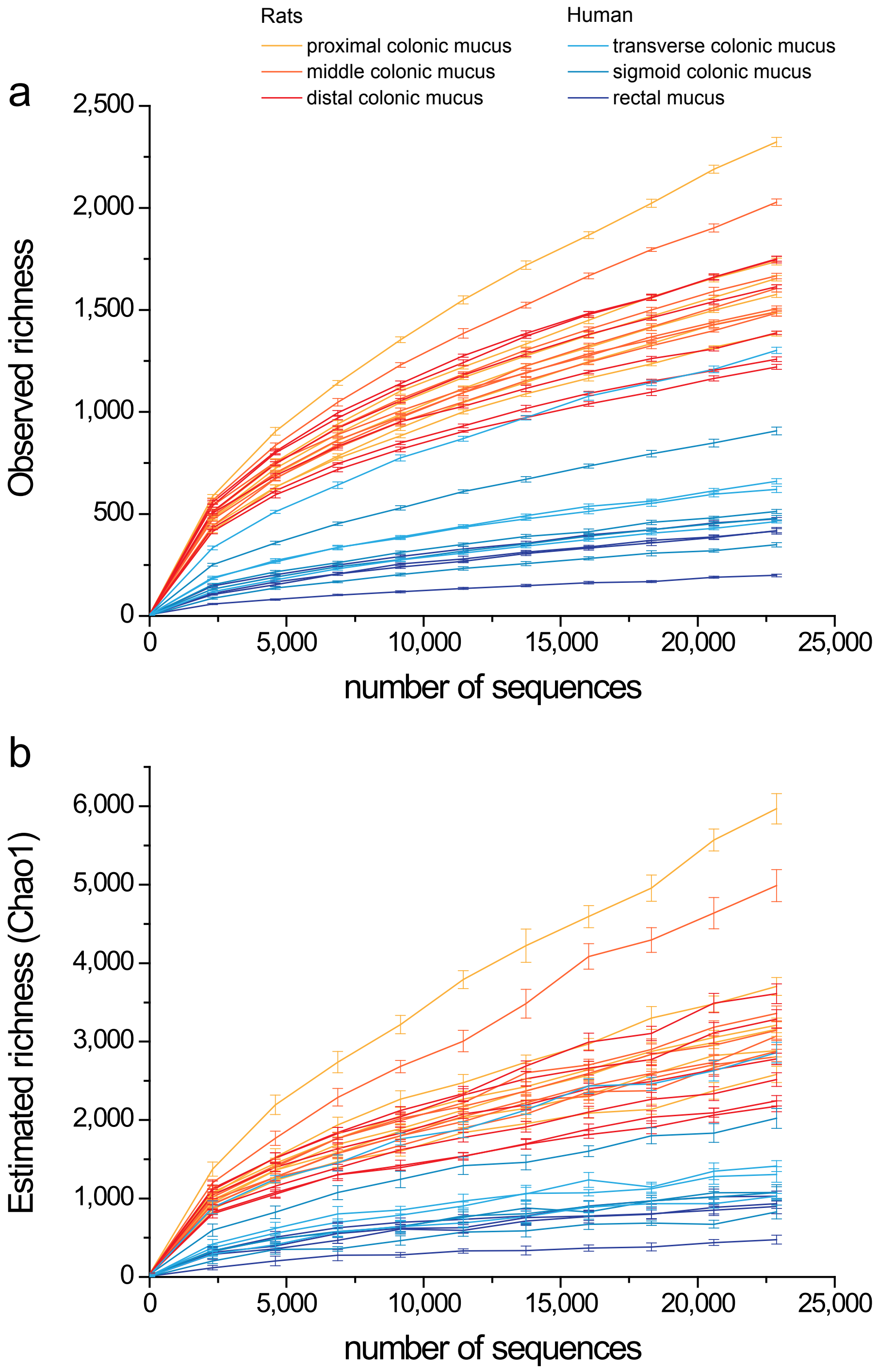

Figure S2. Comparison of rat and human rarefaction curves. (**a**) Observed and (**b**) estimated species richness for samples from rat and human colonic mucus layers. Average OTU richness and Chao1 estimator are presented from ten iterations of rarified subsets of all sequences (on the x-axis) and error bars represent the upper and lower limits of the 95% confidence interval. The human samples were obtained from biopsies of the transverse colon, sigmoid colon and rectum from 4 healthy individuals (reference 14).

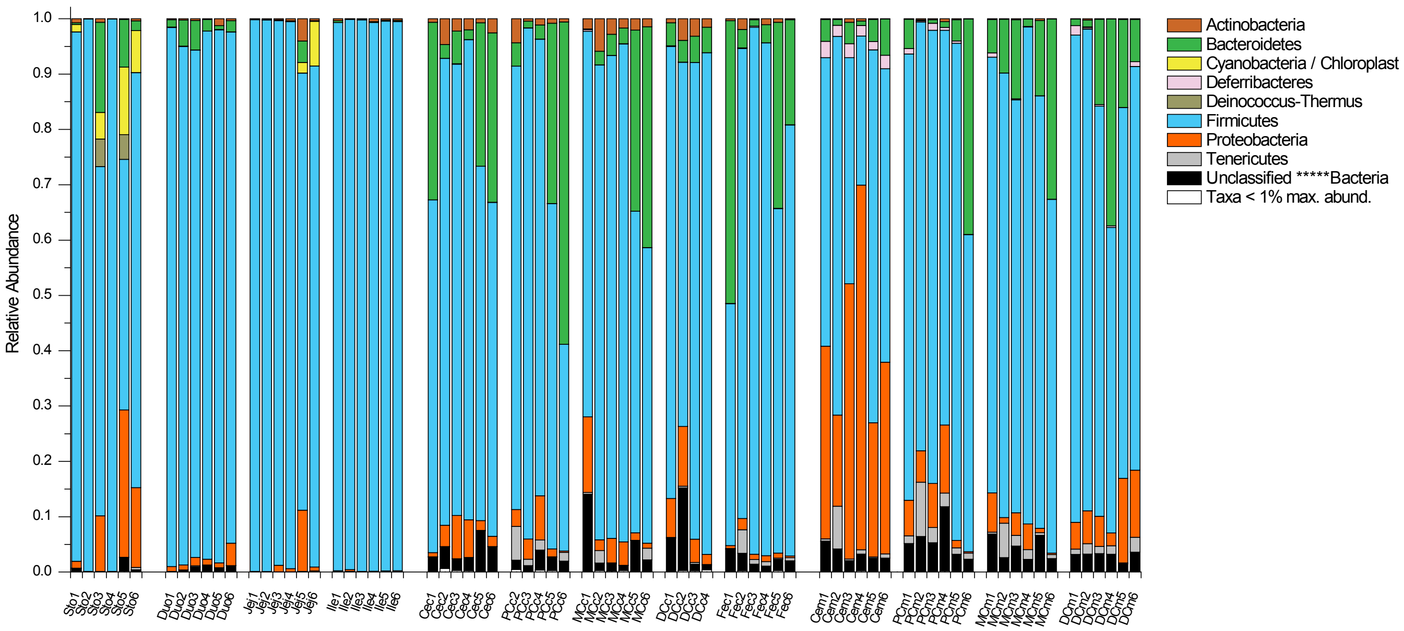

Figure S3. Longitudinal and transverse distribution of dominant taxonomic groups at the phylum level. The relative abundance of different phyla was adjusted by the 16S rRNA copy number data. Sample ID abbreviations are in line with those used in Fig. S1.

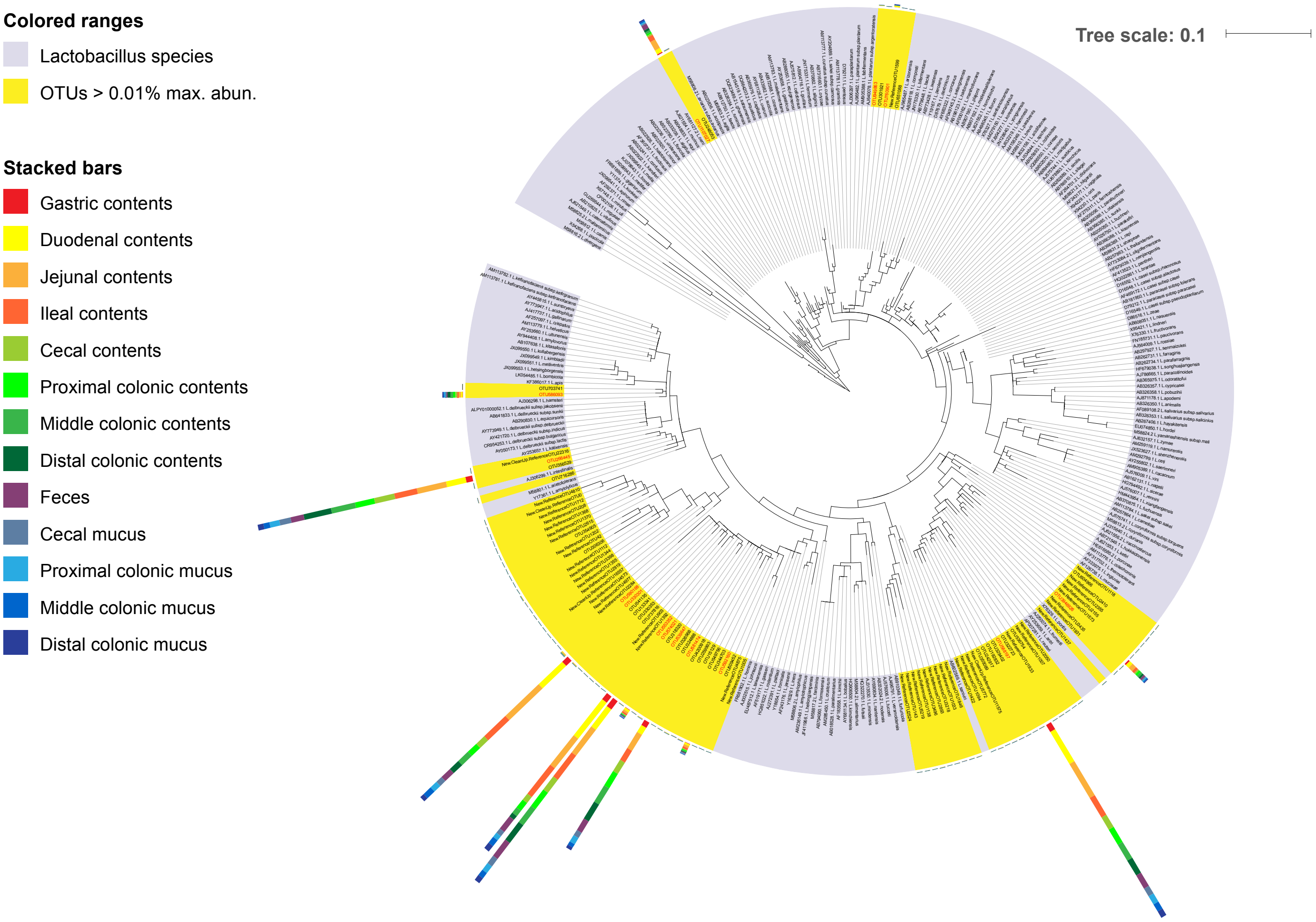

Figure S4. Maximum likelihood tree of all Lactobacillus species and the 90 most abundant Lactobacillus OTUs present along the rat digestive tract. The tree shows the phylogenetic relationship between representative reads of OTUs identified as Lactobacillus and those of Lactobacillus species. The 16S rRNA sequences for the type strain of each species were retrieved from the GenBank database according to the List of Prokaryotic names with Standing in Nomenclature (LPSN, http://www.bacterio.net/). The label for each species node begins with an accession number, followed by the corresponding binomial nomenclature. The heights of stacked bars outside the tree indicate the relative abundances of OTUs at different sampling sites.

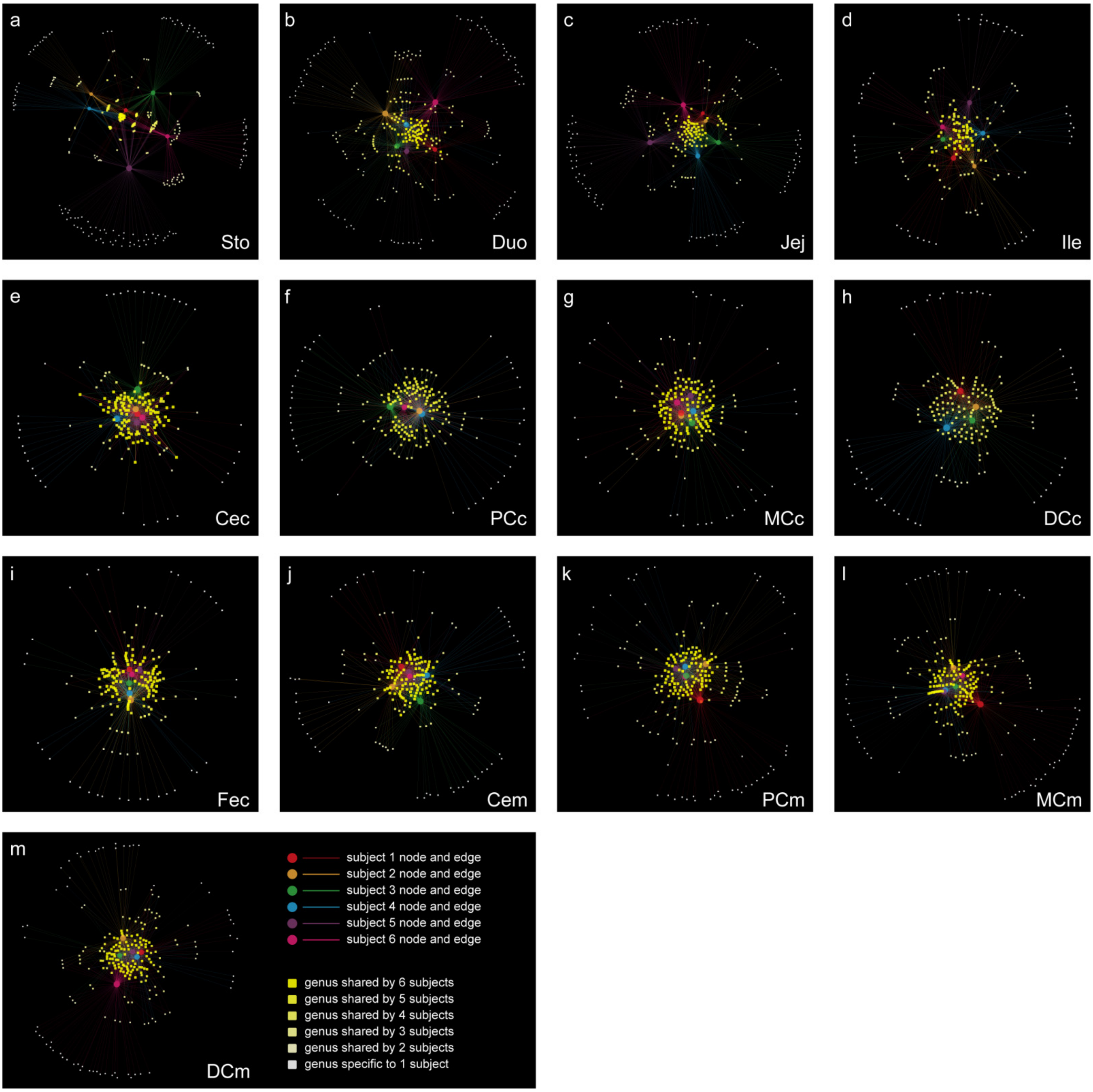

Figure S5. Phylotype (genus) network analysis of microbial communities within different anatomic sites. Bipartite networks were constructed for (**a**) gastric contents, (**b**) duodenal contents, (**c**) jejunal contents, (**d**) ileal contents, (**e**) cecal contents, (**f**) proximal colonic contents, (**g**) middle colonic contents, (**h**) distal colonic contents, (**i**) feces, (**j**) cecal mucus, (**k**) proximal colonic mucus, (**l**) middle colonic mucus and (**m**) distal colonic mucus. Each node represents either a subject or a genus. Connections were drawn between subjects and genera, with edge width in proportion to the abundance of each genus that present in each subject. Node size reflects the degree of one node connected to others.

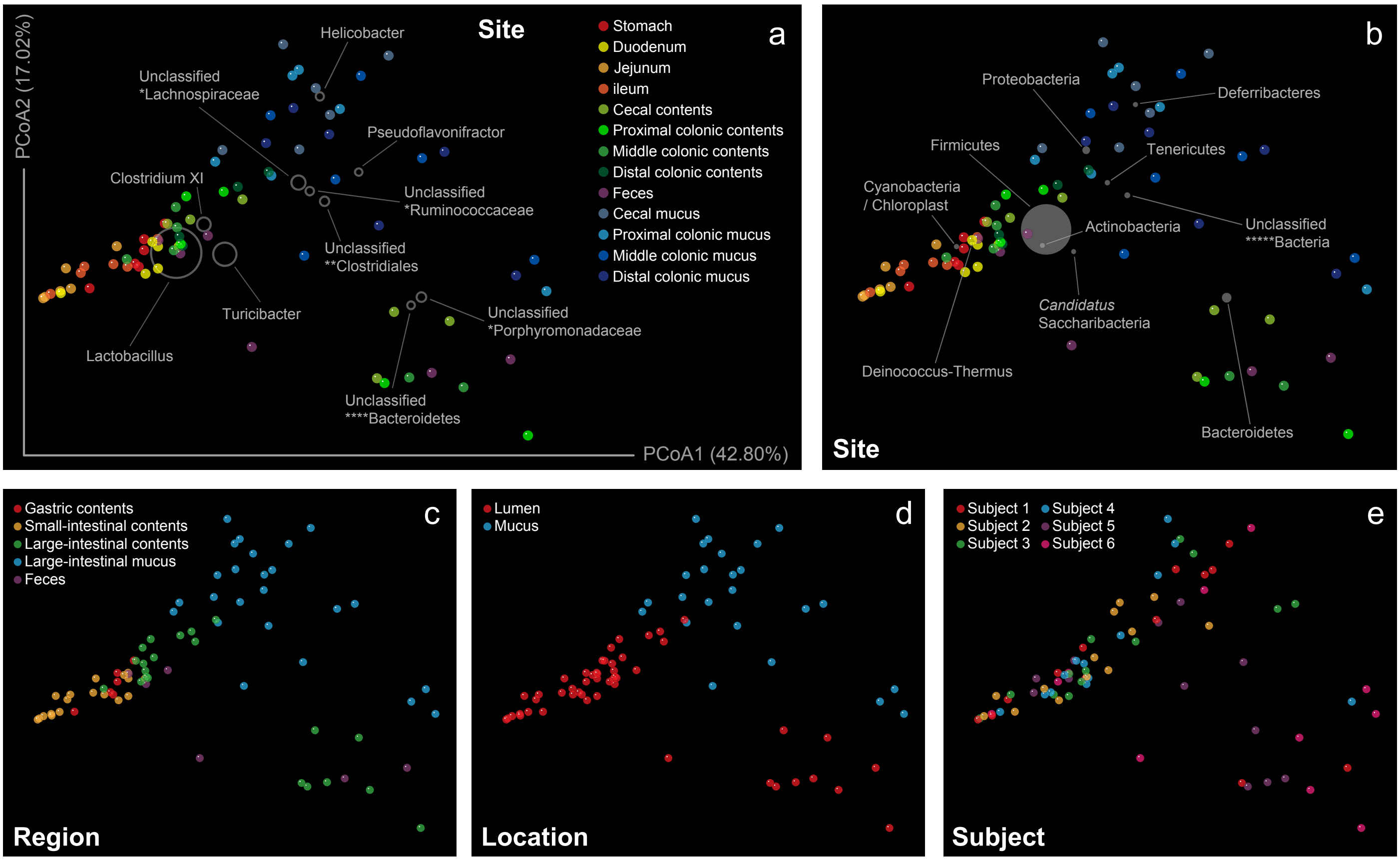

Figure S6. Stratification of the microbial community structure driven by different categorical factors and taxonomic groups. Principal coordinates analysis of weighted UniFrac distances between samples from the rat digestive tract (performed on all operational taxonomic units), grouped by sampling (**a**) site (adonis: R^2 = 0.52; P ≤ 0.001), (**b**) region (adonis: R^2 = 0.43; P ≤ 0.001), (**c**) location (adonis: R^2 = 0.21; P ≤ 0.001) and (**d**) subject (adonis: R^2 = 0.14; P = 0.003). The percent of variation explained by each coordinate is indicated in parentheses. The contribution of different taxonomic groups is represented by the size of the circles (grey) overlayed onto the PCoA based on phylogenetic information. Unclassified genera under a higher rank are marked by asterisks (*family; **order; ***class; ****phylum; *****kingdom).

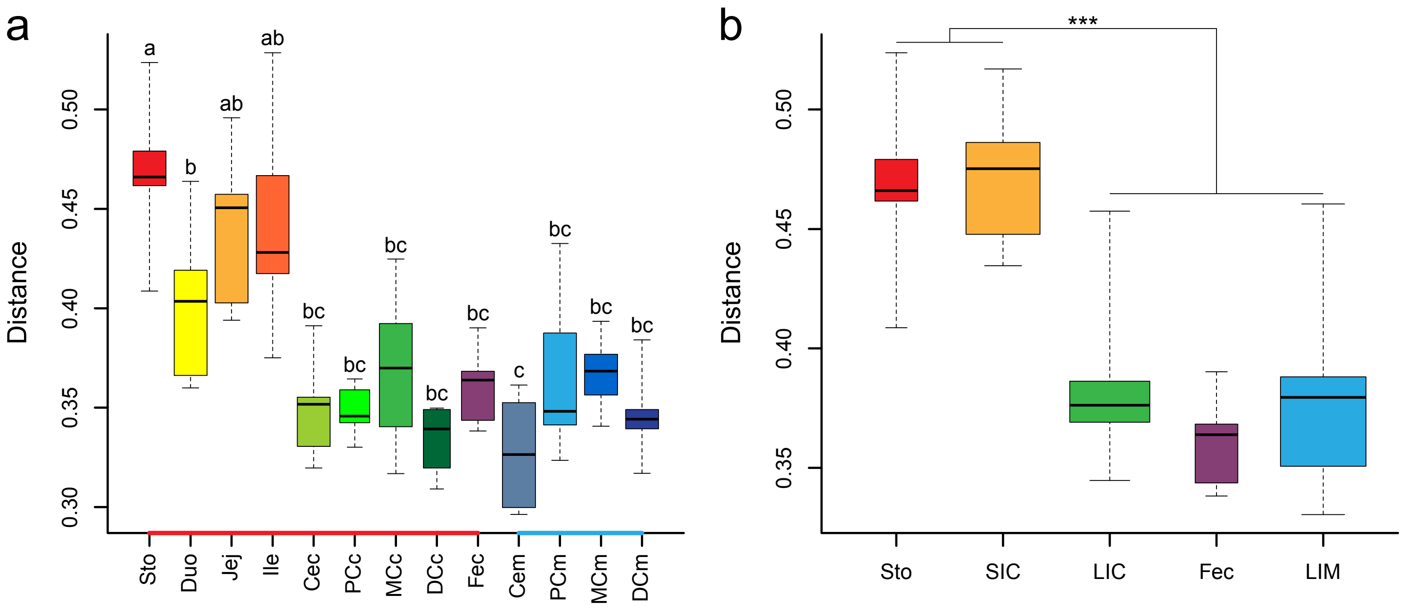

Figure S7. Intra-group dispersions of different anatomic (a) sites and (b) regions in rats. Inter-sample unweighted UniFrac distances were tested for equal intra-group dispersions using betadisper. Data are presented as boxplots with median and min–max whiskers, with box width proportional to the square-root of the number of samples in the group. The significance was calculated using ANOVA with Tukey's HSD *post hoc* test. Sample ID abbreviations are in line with those used in Fig. S1. SIC: small-intestinal contents; LIC: large-intestinal contents; LIM: large-intestinal mucus.

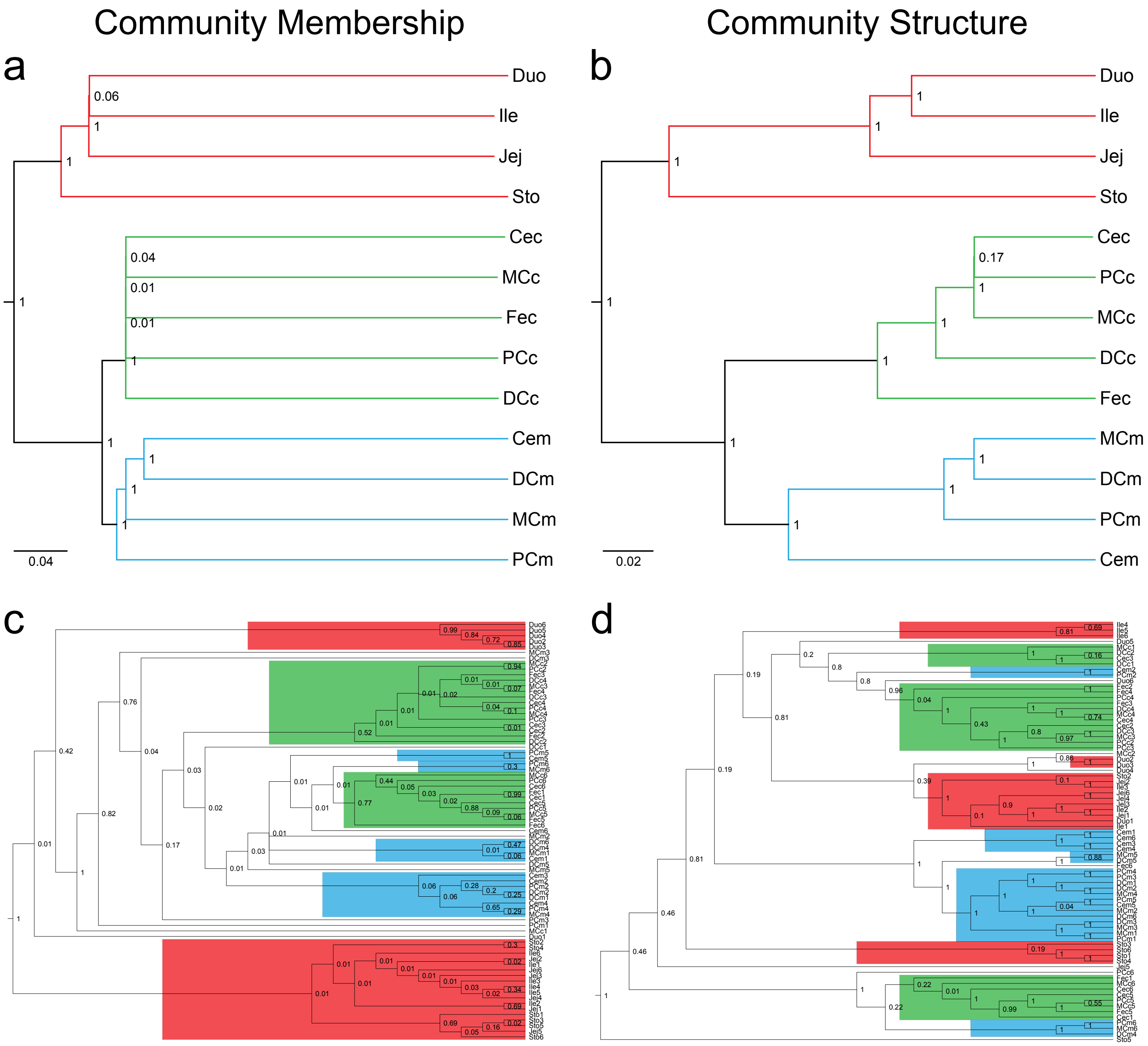

Figure S8. Clustering of 13 anatomic sites and 75 samples according to the microbial community membership and structure. UPGMA trees of (**a**) unweighted and (**b**) weighted UniFrac distances between 13 anatomic sites across subjects. Nodes are labeled with Jackknife values calculated with 100 iterations of 16,000 sequences. UPGMA trees of (**c**) unweighted and (**d**) weighted UniFrac distances between 75 samples from rat digestive tract, with braches transformed as cladograms. Nodes are labeled with Jackknife values calculated with 100 iterations of 18,000 sequences. Sample ID abbreviations are in line with those used in Fig. S1. Branch colours: gastric and small-intestinal contents (red), large-intestinal contents and feces (green), large-intestinal mucus (blue).

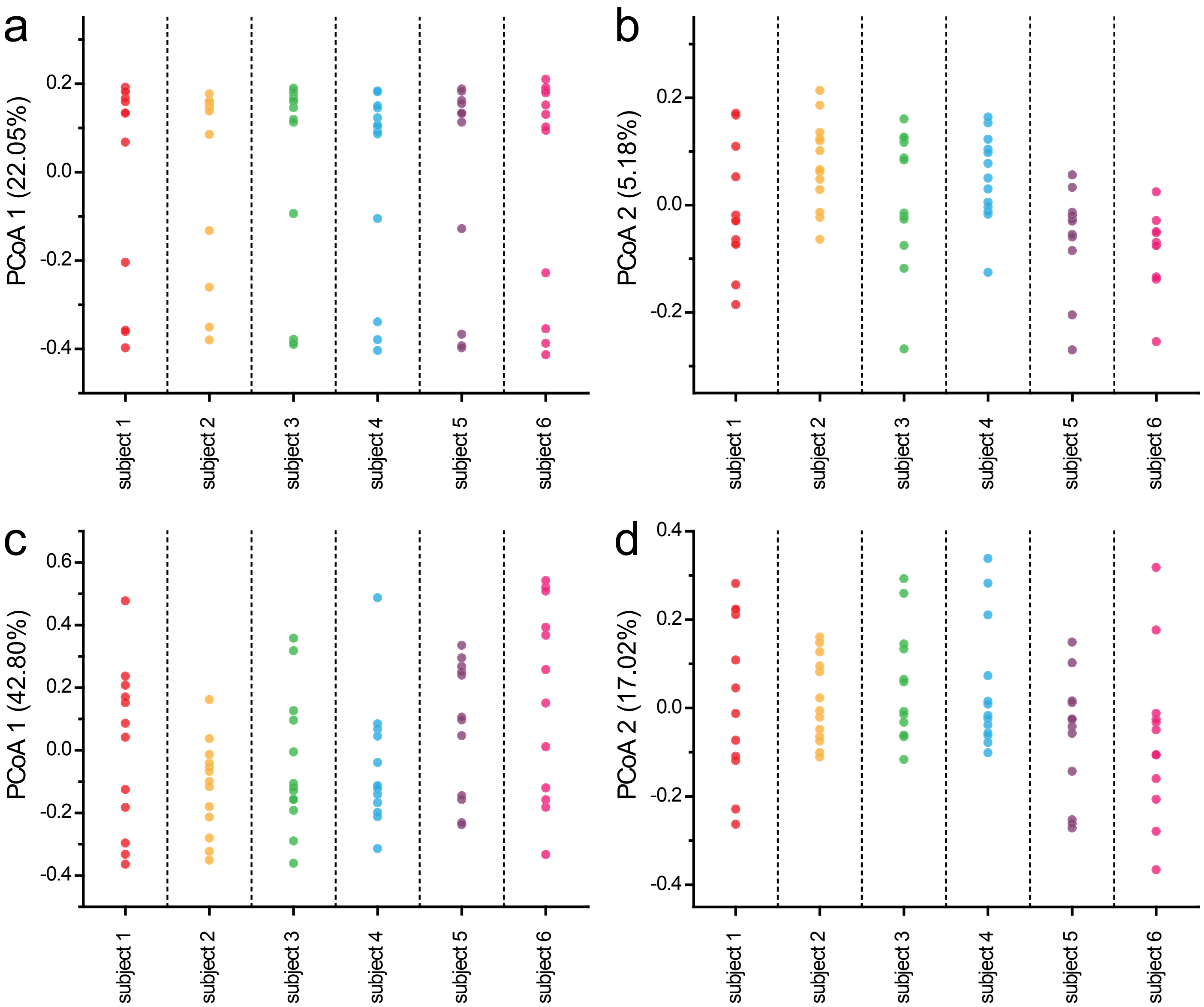

Figure S9. Inter-individual variability of rats depicted by different principal coordinate axes. Plots of (**a**) unweighted UniFrac PCoA1, (**b**) unweighted UniFrac PCoA2, (**c**) weighted UniFrac PCoA1 and (**d**) weighted UniFrac PCoA2 versus subject number.

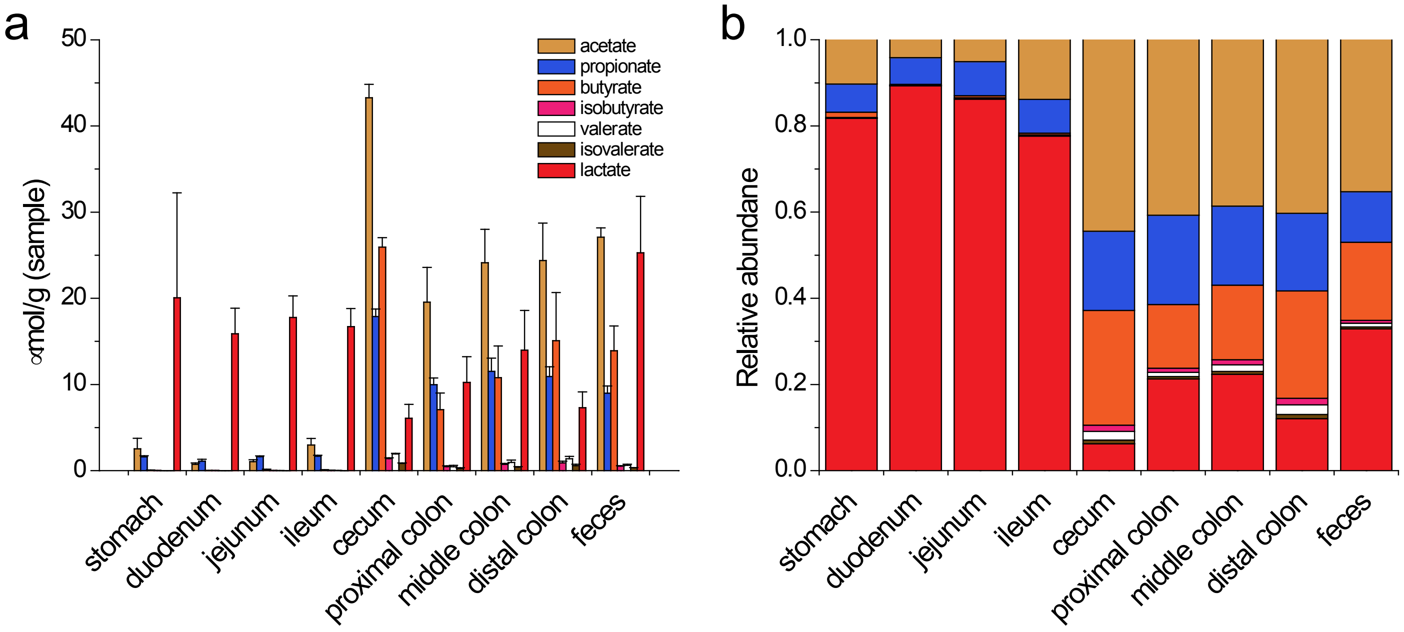

Figure S10. Short-chain fatty acid (SCFA) concentrations in digesta of different gastrointestinal segments. (**a**) Data are presented as mean ± SEM. Significance testing is presented in Table S7. (**b**) Data are presented as relative abundance.

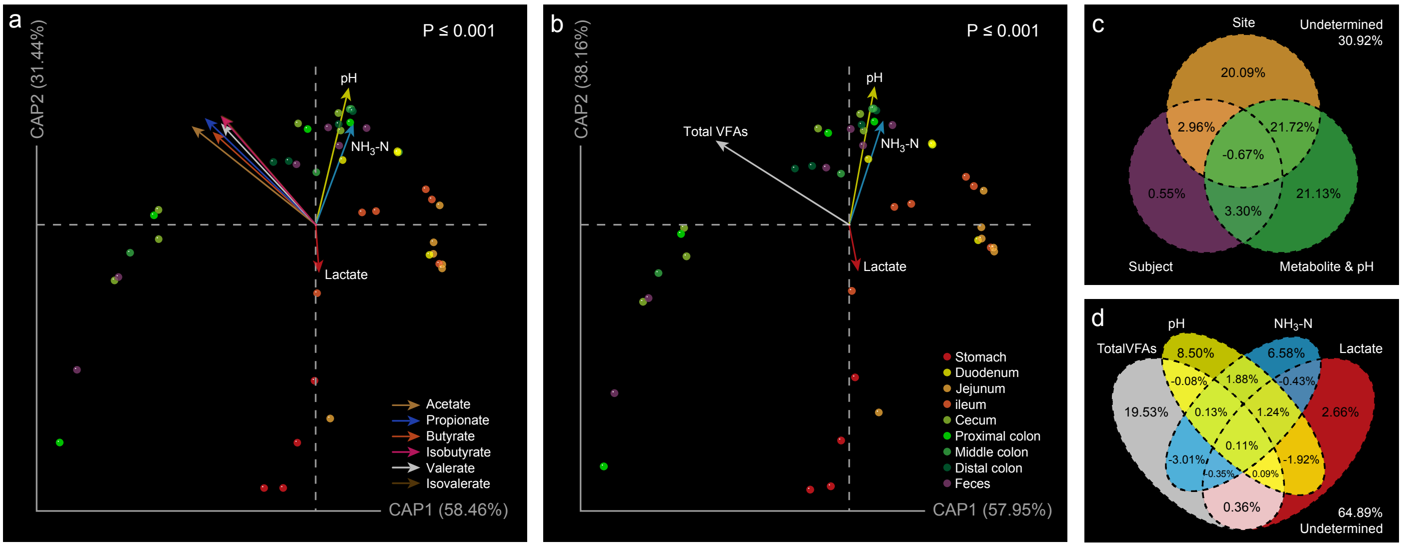

Figure S11. Correlations between environmental variables and microbial community structure. Constrained analysis of principal coordinates of weighted UniFrac distances and environmental variables for luminal samples. Arrows indicate the direction and magnitude of environmental variables associated with bacterial community membership. The P-value of the Monte Carlo permutation test is shown on the upper left. (**a**) Each type of VFA was plotted independently. (**b**) The concentration of several VFAs was summed up and plotted as total VFAs. Venn diagrams demonstrating percentages of community structure variation explained by (**c**) biogeographic location, inter-individual variability, environmental variables and separately explained by (**d**) different environmental variables (pH, total VFAs, lactate and ammonia nitrogen).

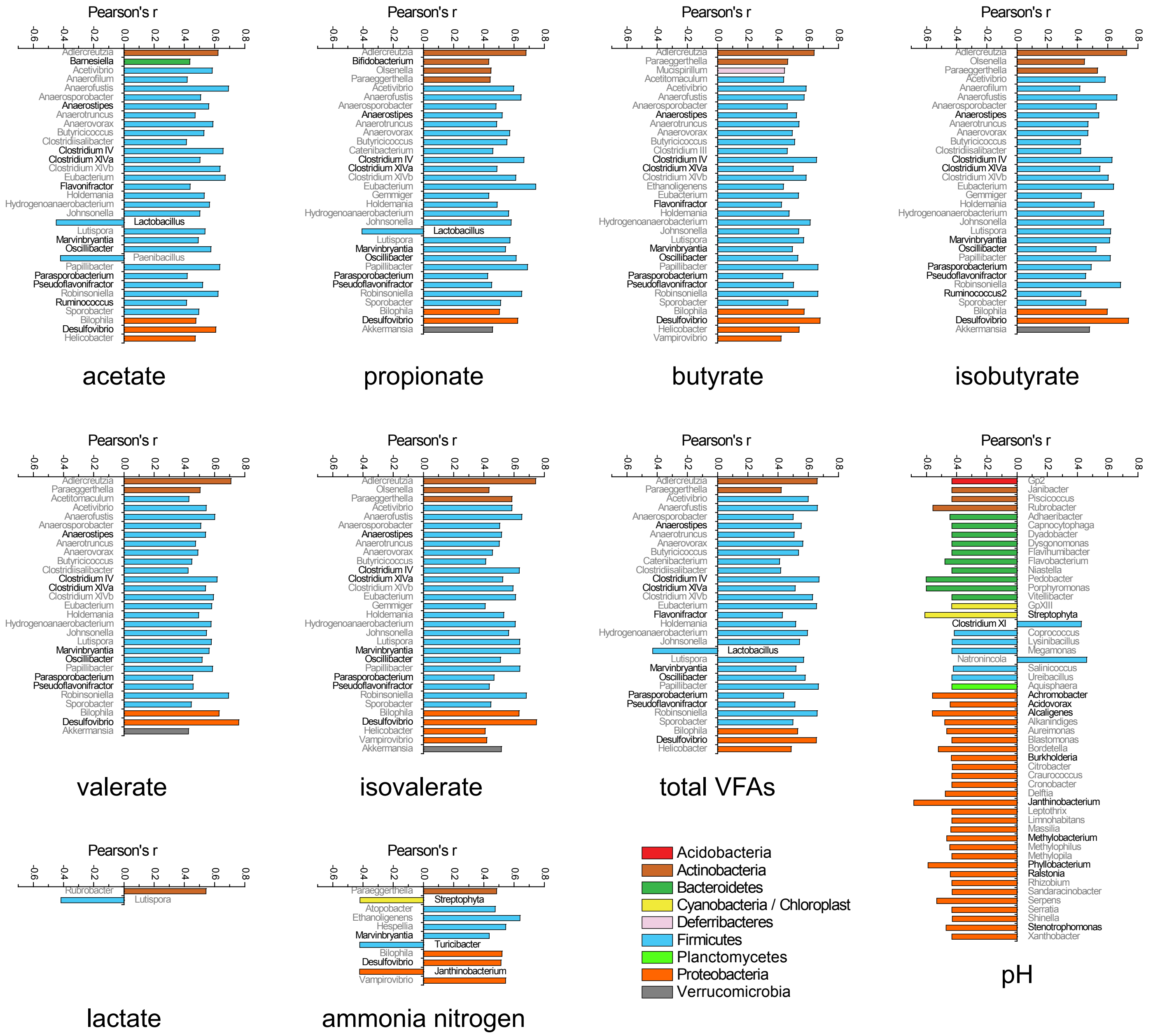

Figure S12. Pearson correlation coefficient plots for different environmental variables. Taxonomic groups at the genus level show different responses to various environmental variables. Plots depict significant (|r| > 0.4 and FDR < 0.05) negative and positive correlations between the relative abundances of particular genera and pH gradients, as well as microbial metabolites. Genera with maximum abundance > 1% are highlighted in black.

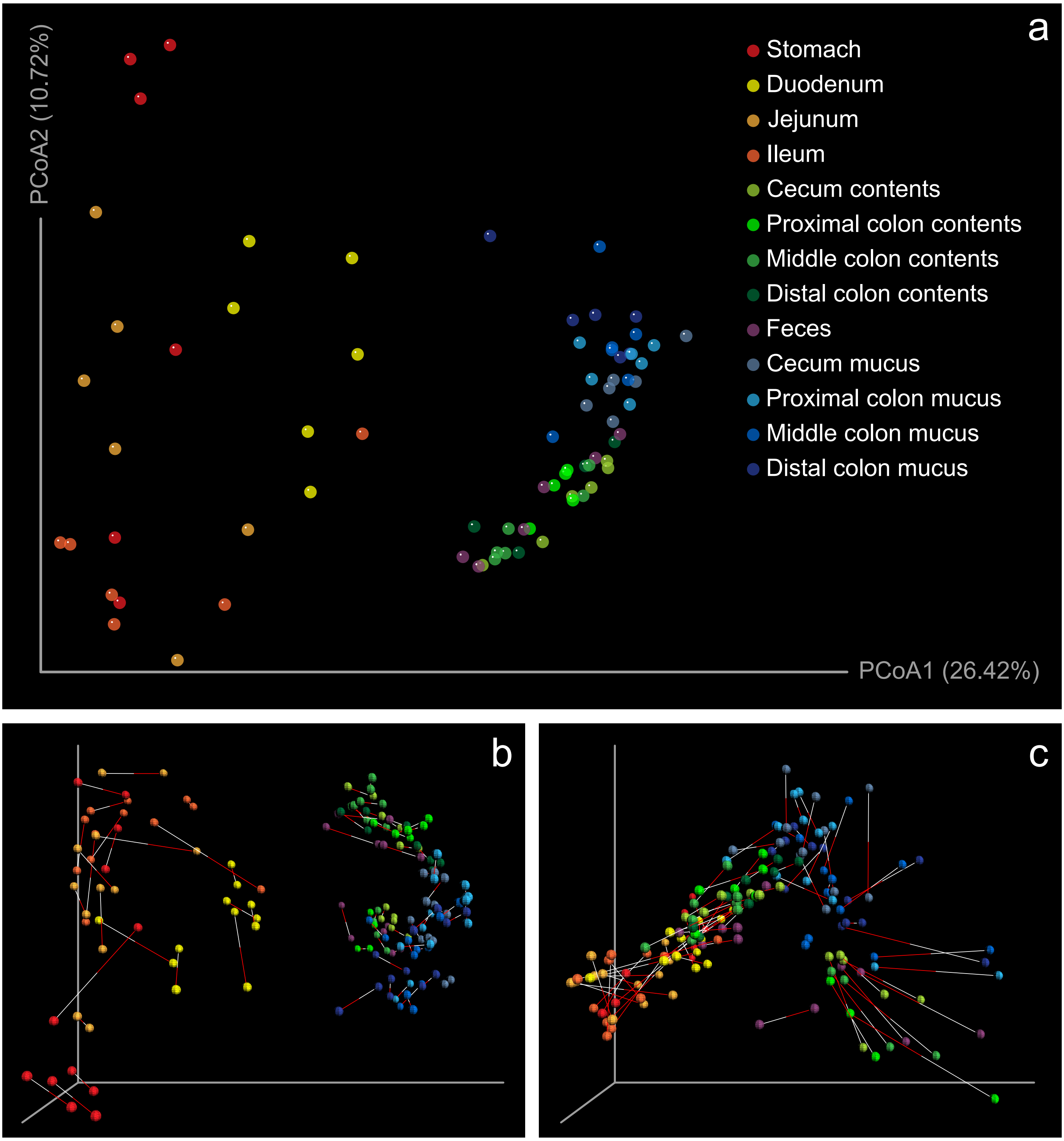

Figure S13. Similarities between different community ordinations. (**a**) Principal coordinates analysis of Bray-Curtis distances between samples from the rat digestive tract (performed on all taxonomic groups at the genus level). Procrustes analyses between the PCoA of (**b**) unweighted UniFrac and Bray-Curtis distances (r = 0.95, P ≤ 0.001), (**c**) weighted UniFrac and Bray-Curtis distances (r = 0.81, P ≤ 0.001).

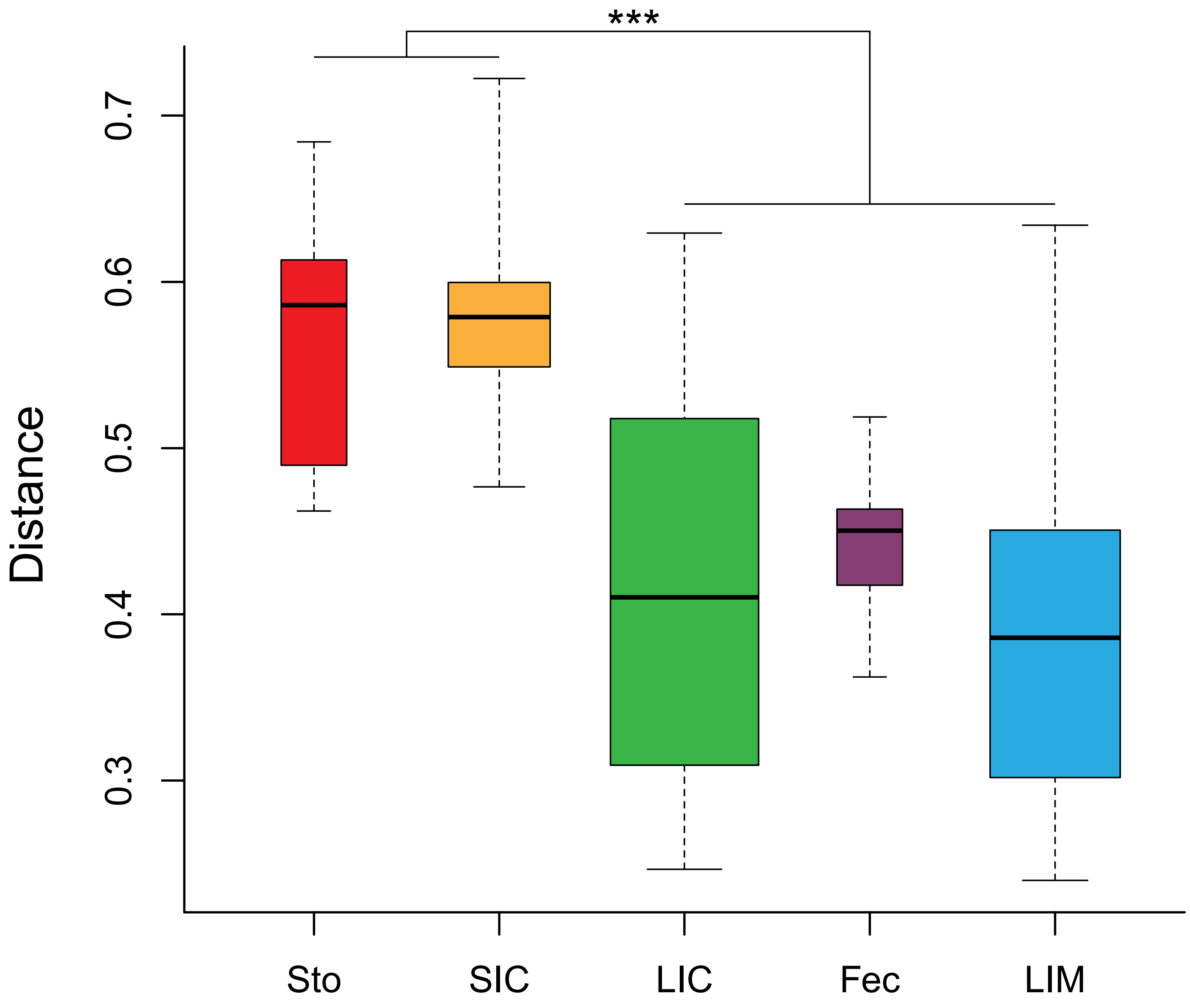

Figure S14. Intra-group dispersions of different anatomic regions in mice, rats and woodrats. Inter-sample Bray-Curtis distances were tested for equal intra-group dispersions using betadisper. Data are presented as boxplots with median and min–max whiskers, with box width proportional to the square-root of the number of samples in the group. The significance was calculated using ANOVA with Tukey's HSD *post hoc* test. SIC: small-intestinal contents; LIC: large-intestinal contents; LIM: large-intestinal mucus.

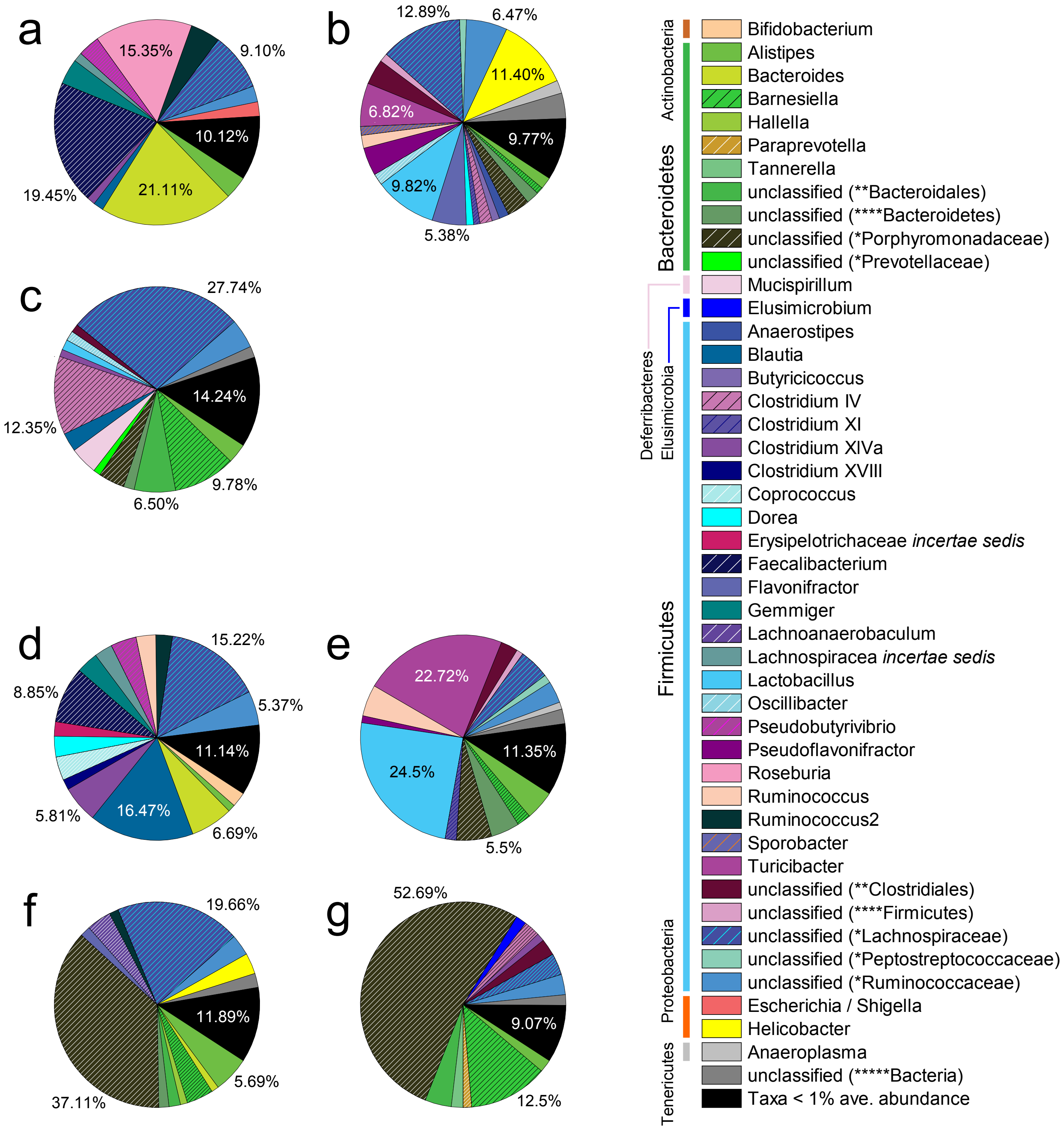

Figure S15. Genus distribution of the large-intestinal mucosal and fecal microbiota in different hosts. The average relative abundance of dominant taxonomic groups at the genus level for (**a**) human colonic and rectal mucus (reference 14), (**b**) rat cecal and colonic mucus (this study), (**c**) mouse cecal and colonic mucus (references 72 and 74), (**d**) human feces (reference 14), (**e**) rat feces (this study), (**f**) mouse feces (reference 57) and (**g**) woodrat feces (reference 73). The abundance of different genera was adjusted by the 16S rRNA copy number data. Unclassified genera under a higher rank are marked by asterisks (*family; **order; ***class; ****phylum; *****kingdom).

Supplementary Tables

Table S1. Length and relative masses (percent of body mass) of various gut chambers in rats. Data are presented as mean ± SEM (n = 6). ND, not determined.

	Stomach	Small intestine			Cecum	Colon
		Duodenum	Jejunum	Ileum		
Length (cm)	ND		67.83±1.35		ND	11.67±0.33
Percent of body mass (%)	1.04±0.11	0.55±0.02	1.80±0.12	1.33±0.07	1.83±0.09	1.04±0.09

Table S2. Overview of sequencing results for each sample. The sequence number refers to the count of assembled sequences after quality filtering. The OTU number, Chao1 estimator and Good's coverage are presented for all sequences without rarefaction. Sample ID abbreviations are in line with those used in Fig. S1.

Sample ID	Sequence Number	OTU Number	Chao1	Good's coverage
Sto1	31,794	461	1,074.68	0.9917
Sto2	52,937	798	1,925.98	0.9913
Sto3	22,887	452	984.34	0.9891
Sto4	38,336	554	1,475.81	0.9910
Sto5	54,484	1,046	2,682.25	0.9891
Sto6	31,842	434	981.50	0.9931
Duo1	39,627	706	1,579.98	0.9897
Duo2	53,698	1,305	2,491.44	0.9883
Duo3	69,474	1,482	2,989.70	0.9895
Duo4	47,478	1,272	2,757.19	0.9859
Duo5	50,854	1,477	3,684.14	0.9838
Duo6	57,710	1,360	3,406.81	0.9874
Jej1	47,845	555	1,165.51	0.9931
Jej2	47,184	781	1,902.76	0.9901
Jej3	77,684	1,213	2,978.07	0.9908
Jej4	52,669	978	2,343.28	0.9887
Jej5	63,174	906	2,127.63	0.9920
Jej6	41,823	842	1,930.16	0.9882
Ile1	76,060	1,873	4,668.17	0.9857
Ile2	29,554	501	1,214.33	0.9891
Ile3	52,762	1,047	2,512.45	0.9884
Ile4	36,139	662	1,649.54	0.9891
Ile5	36,925	873	2,029.91	0.9863
Ile6	31,361	537	1,530.05	0.9893
Cec1	47,659	1,763	3,394.41	0.9807
Cec2	55,722	2,699	6,621.55	0.9717
Cec3	104,844	4,066	10,554.62	0.9775
Cec4	62,352	2,421	6,310.95	0.9774

Cec5	53,158	2,369	5,393.61	0.9761
Cec6	45,373	2,088	4,720.22	0.9761
PCc2	60,869	2,578	6,137.38	0.9761
PCc3	195,780	5,536	14,494.52	0.9837
PCc4	67,262	2,987	6,988.76	0.9752
PCc5	51,896	2,096	4,476.71	0.9786
PCc6	41,879	1,506	3,204.98	0.9808
MCc1	55,370	2,009	4,671.94	0.9791
MCc2	43,689	1,660	3,662.74	0.9789
MCc3	57,609	2,198	5,050.11	0.9788
MCc4	50,783	2,274	5,263.20	0.9750
MCc5	65,651	2,378	5,587.01	0.9800
MCc6	62,340	2,359	4,822.44	0.9798
DCc1	33,885	2,231	5,323.76	0.9631
DCc2	34,906	2,025	4,429.28	0.9682
DCc3	80,893	3,119	6,970.61	0.9785
DCc4	57,266	2,204	4,887.56	0.9787
Fec1	48,899	2,038	4,453.58	0.9778
Fec2	44,959	2,660	6,699.78	0.9649
Fec3	40,080	1,881	4,104.37	0.9741
Fec4	34,528	1,686	3,958.03	0.9716
Fec5	84,133	2,537	5,614.72	0.9843
Fec6	43,328	1,546	3,184.14	0.9813
Cem1	37,661	1,649	2,964.36	0.9803
Cem2	51,312	2,354	5,002.98	0.9755
Cem3	70,297	2,340	4,288.25	0.9844
Cem4	47,459	1,703	3,494.08	0.9816
Cem5	39,192	2,010	3,953.37	0.9745
Cem6	29,273	1,579	2,804.03	0.9760
PCm1	110,053	6,005	16,750.71	0.9658
PCm2	44,315	2,084	4,299.51	0.9762
PCm3	85,176	3,513	8,889.25	0.9770
PCm4	51,949	2,397	5,706.61	0.9759
PCm5	51,677	2,458	4,916.85	0.9766
PCm6	43,688	1,860	3,705.62	0.9802
MCm1	63,551	2,367	5,422.19	0.9819
MCm2	48,980	2,146	4,577.13	0.9779
MCm3	46,620	3,048	8,421.03	0.9615
MCm4	52,879	2,506	5,317.79	0.9766
MCm5	39,879	2,149	4,598.29	0.9714
MCm6	43,945	2,055	4,092.50	0.9778
DCm1	43,109	2,339	4,715.11	0.9746
DCm2	43,426	2,137	4,321.92	0.9778
DCm3	36,016	2,205	5,524.35	0.9682
DCm4	46,593	1,706	3,434.33	0.9837
DCm5	54,921	1,793	3,411.05	0.9853
DCm6	57,774	2,091	4,885.96	0.9827

Table S3. The 16S rRNA copy number adjusted counts for genera present in each sample. Taxonomy is based on the RDP classifier trained with the 16S rRNA training set No. 14 with a bootstrap cutoff of 50%. See Supplemental_Table_S3.xlsx.

Table S4. Significantly different phyla and genera in anatomic sites along rat alimentary canal. Statistical tests of over- or under-representation of bacterial lineages amongst anatomic sites were made at the phylum and genus levels using Kruskal-Wallis rank sum test. P-values were adjusted for multiple testing by false discovery rate. The direction column sorts sites by their mean abundance, from largest to smallest. "-" corresponds to no sequences present. VIP, Variable importance in projection. See Supplemental_Table_S4.xlsx.

Table S5. Core taxonomic groups shared within different anatomical sites at the OTU and genus level. “Core” columns refer to the number of taxonomic groups shared by all subjects, while “full” columns refer to the number of all taxonomic groups found in a given anatomic site.

	Site	OTU			genus		
		core	full	[%] core / full	core	full	[%] core / full
Contents	Stomach	36	2,526	1.43%	16	259	6.18%
	Duodenum	149	4,323	3.45%	51	275	18.55%
	Jejunum	70	3,195	2.19%	31	235	13.19%
	Ileum	70	3,338	2.10%	31	160	19.38%
	Cecum	475	8,290	5.73%	92	172	53.49%
	Proximal colon	506	8,805	5.75%	83	188	44.15%
	Middle colon	352	6,839	5.15%	79	178	44.38%
	Distal colon	627	5,801	10.81%	96	176	54.55%
	Feces	353	6,510	5.42%	75	170	44.12%
Mucus	Cecum	437	5,575	7.84%	87	195	44.62%
	Proximal colon	533	10,577	5.04%	78	215	36.28%
	Middle colon	380	7,866	4.83%	80	216	37.04%
	Distal colon	380	6,314	6.02%	82	228	35.96%

Table S6. List of sequence counts for 97% OTU clusters shared across all subjects and all sampling regions. The “full” row at the end of the table lists the counts of all sequences for given sampling regions. Close reference OTU ID is based on greengenes database (release 13.8). Taxonomy is based on the RDP classifier trained with the 16S rRNA training set No. 14 via a bootstrap cutoff of 50%. See Supplemental_Table_S6.xlsx.

Table S7. pH and concentrations of microbial metabolite (μmol/g sample) along the length of the rat digestive tract. Means are listed, with ranges shown in parentheses. P-values for the Kruskal-Wallis rank sum test were corrected by false discovery rate. Different superscript letters (a, b) mean significant differences between groups (Nemenyi's *post hoc* multiple comparison test, FDR <0.05). See Supplemental_Table_S7.xlsx.
